# The gut microbiota mediates protective immunity against tuberculosis *via* modulation of lncRNA

**DOI:** 10.1080/19490976.2022.2029997

**Published:** 2022-03-28

**Authors:** Fang Yang, Yi Yang, Lingming Chen, Zhiyi Zhang, Linna Liu, Chunmin Zhang, Qiongdan Mai, Yiwei Chen, Zixu Chen, Tao Lin, Liang Chen, Huixin Guo, Lin Zhou, Hongbo Shen, Xinchun Chen, Lei Liu, Guoliang Zhang, Hongying Liao, Lingchan Zeng, Gucheng Zeng

**Affiliations:** a Department of Microbiology Zhongshan School of Medicine, Key Laboratory for Tropical Diseases Control of the Ministry of Education, Sun Yat-sen University, Guangzhou China; b Drepartment of Pediatric Intensive Care Unit, Guangzhou Women and Children’s Medical Center, Picu, Guangzhou China; c Guangdong Center for Tuberculosis Control, National Clinical Research Center for Tuberculosis, Guangzhou China; d Clinic and Research Center of Tuberculosis, Shanghai Key Laboratory of Tuberculosis, Shanghai Pulmonary Hospital, Institute for Advanced Study, Tongji University School of Medicine, Shanghai, China; e Department of Pathogen Biology, Shenzhen University School of Medicine, Shenzhen, Guangdong China; f Institute for Hepatology, National Clinical Research Center for Infectious Disease, Guangdong Key Laboratory for Emerging Infectious Diseases, Shenzhen Third People’s Hospital, National Clinical Research Center for Tuberculosis, Southern University of Science and Technology, National Clinical Research Center for Tuberculosis, Shenzhen, China; g De partment of Thoracic Surgery, Thoracic Cancer Center, the Sixth Affiliated Hospital of Sun Yat-sen University, Guangzhou, China; h Clinical Research Center, Department of Medical Records Management, Guanghua School of Stomatology, Hospital of Stomatology, Sun Yat-sen University, Guangzhou, Guangdong, China

**Keywords:** Tuberculosis, gut-lung axis, commensal gut bacteria

## Abstract

The gut-lung axis has been implicated as a potential therapeutic target in lung disorders. While increasing evidence suggests that gut microbiota plays a critical role in regulating host immunity and contributing to tuberculosis (TB) development and progression, the underlying mechanisms whereby gut microbiota may impact TB outcomes are not fully understood. Here, we found that broad-spectrum antibiotics treatment increased susceptibility to *Mycobacterium tuberculosis* (*M. tuberculosis*) infection and modulated pulmonary inflammatory responses in mouse *M. tuberculosis* infection model. We then identified a commensal gut bacteria-regulated lncRNA, termed lncRNA-CGB, which was down-regulated by dysbiosis of gut microbiota during TB infection. Furthermore, we found that *Bacteroides fragilis* (*B. fragilis*) was a direct regulator of lncRNA-CGB, and oral administration of *B. fragilis* enhanced expression of lncRNA-CGB and promoted anti-TB immunity. Genomic knock-out of lncRNA-CGB led to reduced IFN-γ expression and impaired anti-TB immunity, therefore leading to detrimental effects on *M. tuberculosis* infection. Mechanistically, lncRNA-CGB interacted with EZH2 and negatively regulated H3K27 tri-methylation (H3K27Me3) epigenetic programming, leading to enhanced IFN-γ expression. Thus, this work not only uncovered previously unrecognized importance of gut bacteria-lncRNA-EZH2-H3K27Me3 axis in conferring immune protection against TB but also identified a potential new paradigm to develop a microbiota-based treatment against TB and potentially other diseases.

## Introduction

The gastrointestinal tract represents a large ecosystem, housing millions of microbial cells, which are of critical importance for modulating systemic equilibrium of the immune system toward healthy homeostasis.^1,2^ The concept of the gut-lung axis was born out of the observation that different lung diseases could be influenced by intestinal microenvironment changes and vice versa.^1,2^ Moreover, a higher prevalence of pulmonary diseases in those patients who also have chronic gastrointestinal diseases highlights the importance of crosstalk between the gut and lung.^3^ Increasing evidence has highlighted that disturbing the gut-lung axis may contribute to impaired lung homeostasis and influencing the outcomes of lung diseases, such as asthma, chronic obstructive pulmonary disease, respiratory virus infection, and cystic fibrosis.^4–8^

*Mycobacterium tuberculosis* (*M. tuberculosis*) infects one-third of the world population and leads to tuberculosis (TB), a deadly infectious disease with currently an estimated 1.4 million deaths annually.^9,10^ While the complex interplay between genetic factors, host- and pathogen-specific factors contribute to the development and progression of the TB infection, significant risk factors for developing active TB are associated with both structural and functional changes in the gut microbiota.^11–13^ Emerging evidence^14,15^ has suggested that altered microbiota is likely to have deleterious consequences for susceptibility to TB development and progression. Successfully addressing TB infection and disease may require targeting other modifiable risk factors, such as host microbiota. However, available research findings provide relatively limited evidence on the crosstalk between gut and lung during TB infection, and the underlying mechanism(s) whereby gut microbiota regulates the TB disease development and progression are largely not explained.

It has been suggested that the soluble microbial components and metabolites transported *via* the circulation are one means of communication between the gut and the lung.^1,2^ The gut microbiota therefore can directly and/or indirectly influence lung homeostasis *via* host-derived inflammatory mediators and determine the protective or pathological outcomes of microbial infection not only in gastrointestinal tract but also in lungs.^16–20^ For instance, major mechanisms attributed to the protective effect of lactobacilli during respiratory infection are the induction of protective immunity *via* stimulation of the immunomodulatory mediators such as IFN-γ as well as the inhibition of lung immunopathology *via* the induction of IL-10.^21^ However, the signals from the gut microbiota that affect TB infections in lungs are largely undetermined.

While the roles of protein-coding genes in host-microbiota interactions have been subjected to intensive investigations, emerging evidence has revealed that regulatory non-coding RNAs (ncRNAs) play a critical role in modulating host-microbe interactions, and ncRNAs have been proposed as potential modulators of the host response to microbiome-linked pathologies such as cancers and obesity.^22^ An in-depth understanding of the roles and underlying mechanisms of ncRNAs in modulating host-microbe interactions involving gut-lung axis thus may provide more effective diagnostic tools and therapeutic agents for microbiome-linked diseases not only locally but also systemically. Recent studies have reported that compared with germ-free mice, those that were colonized with specific bacteria displayed a significantly different long non-coding RNA (lncRNA) profile, which suggest that crosstalk between host microbiota and noncoding RNAs may play a role in TB development, progression and prognosis.^22,23^ However, little is known as to whether and by which gut microbiota modulate circulating non-coding RNAs such as lncRNAs that contribute to *M. tuberculosis* infection outcomes.

Significant amounts of lncRNAs have been identified in the mammalian genomes,^24^ of which only a minority, but increasingly more, have been functionally characterized in different processes, including infections.^25,26^ Some host lncRNAs have been reported to regulate the expression of inflammatory mediators or be regulated by interferon (IFN) in respiratory viral infection,^27^ and our previous identification of lncRNA-CD244 suggested that lncRNA might have immunomodulatory properties for a more effective TB control.^28^ However, whether a specific lncRNA is involved in gut-lung axis during TB infection in response to gut microbiota-derived cues is unclear. Thus, understanding how the intestinal microbiota primes the host inflammatory response through regulating the expression and functions of lncRNA to combat TB is vital for shaping future strategies of more effective prevention and treatment of TB.

Here, we found that dysbiosis of gut microbiota induced more severe TB infection and identified a series of commensal bacteria-associated lncRNAs, of which, a lncRNA (lncRNA-ENSMUSG00000086503, termed lncRNA-CGB) was significantly down-regulated in *M. tuberculosis*-infected mice with disruption of gut bacteria. Consistently, the human homologue of lncRNA-CGB was further characterized as the most significantly depressed lncRNA in active TB patients whose gut microbiome’s diversity was perturbed. Genomic knock-out of lncRNA-CGB developed much more severe TB disease after *M. tuberculosis* infection hinting that lncRNA-CGB played a beneficial role in developing immune resistance to *M. tuberculosis* infection and TB pathology. Moreover, we found that gut dysbiosis depleted bacterial species regulating proper immune functioning and therefore induced significantly repressed *M. tuberculosis*-killing cytokines, IFN-γ. Furthermore, oral transfer of the resident intestinal probiotic *Bacteroides fragilis (B. fragilis)* sustained expression of lncRNA-CGB, and promoted anti-TB immune protection *via* epigenetically modulating IFN-γ expression, indicating that a selected lncRNA signature modulated by commensal gut bacteria might be a key mediator regulating gut-lung axis homeostasis during *M. tuberculosis* infection.

Thus, this work not only uncovered previously unrecognized importance of gut microbiota-dictated lncRNA signature in conferring immune protection in gut-lung axis but also highlighted a potentially new paradigm to develop microbiota-based therapeutic interventions for lung diseases.

## Results

### Gut dysbiosis disrupts lung homeostasis and promotes M. tuberculosis infections in M. tuberculosis-infected mouse model

**Figure 1.**
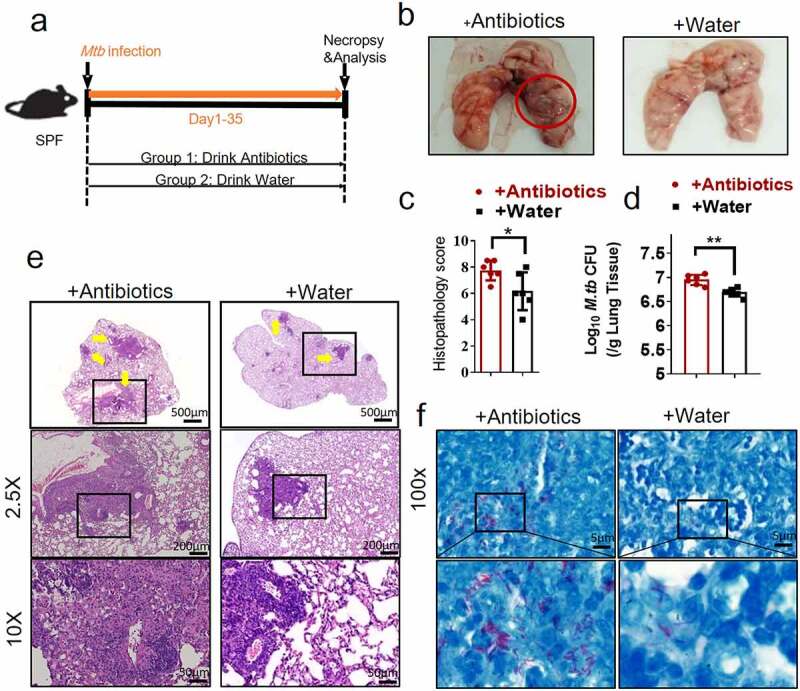
Gut microbiota is required for the control of *M. tuberculosis* infection.

As an initial step to determine whether gut microbiota regulated the *M. tuberculosis* outcomes through modulating the expression and function of the lncRNAs, we first analyzed whether disruption of gut microbiota *via* antibiotics feeding could impact TB infection outcomes, thus we performed an antibiotic administration protocol during TB infection. Rapid loss of diversity of gut microbiota was observed after the broad-spectrum antibiotics (ampicillin, neomycin, vancomycin and metronidazole) feedings (Supplementary Figure S1). More importantly, oral treatment of these antibiotics was found to induce dysbiosis, but no significant effect on *M. tuberculosis* viability was observed even in mice with antibiotics administration for 42 days.^29^ To this end, mice were fed daily with broad-spectrum antibiotics or saline as control during *M. tuberculosis* infection (Figure 1a). Therefore, the TB infection outcomes with or without the disruption of bacterial equilibrium by antibiotics treatment in mice can be determined. When compared with water controls, antibiotics feedings led to more severe lung pathology characterized by larger scale damage of lung structure and defined granuloma, more significant infiltration of inflammatory cells in the lungs (Figure 1b, c, e), much higher *M. tuberculosis* burdens in pulmonary compartments (Figure 1d), and more bacilli positive for acid-fast staining in granuloma lesion area, compared with water controls (Figure 1f). Thus, these results not only suggested that microbial diversity in the gastrointestinal tract was required to prime or sustain systemic immune resistance toward TB but also outlined the potential role of the gut microbiota in gut-lung crosstalk involved in TB infection.

(a) Experimental design to test the importance of gut microbiota in control of *M. tuberculosis* infection and TB pathology. *M. tuberculosis*-infected mice were treated with sterilized water only or with broad-spectrum antibiotics (ampicillin (1 mg/ml), neomycin (1 mg/ml), metronidazole (1 mg/ml) and vancomycin (0.5 mg/ml)) in their drinking water until mice were sacrificed for immunological, pathological, and bacillus burden analyses. (b) Gross pathology of representative lungs of *M. tuberculosis*-infected mice with or without drinking broad-spectrum antibiotics. More severe lesions were marked with red circles. (c) Blinded scoring of H&E staining of lung sections derived from mice with or without drinking antibiotics. (d) *M. tuberculosis* CFU quantitative analyses in the lungs (per gram) in *M. tuberculosis*-infected mice with or without drinking antibiotics. (e) H&E staining of representative lungs of *M. tuberculosis*-infected mice with or without drinking broad-spectrum antibiotics. Yellow arrows marked extensive damages of pulmonary structures and extensive infiltration of inflammatory cells in the pulmonary compartments observed in the lung sections derived from *M. tuberculosis*-infected mice. (f) Acid-fast staining of lung sections derived from *M. tuberculosis*-infected mice with or without drinking antibiotics. *M. tuberculosis* bacilli in granuloma lesions highlighted in the lower panel. Note that more bacilli positive for acid-fast staining were observed in lesions of *M. tuberculosis*-infected mice with drinking antibiotics. Lower panel: higher-resolution view of the regions in the box. N = 6 SPF mice for each group of mice for each experiment, and results are representative of three independent experiments. Error bars indicate average values ± SEM, *p < .05 **p < .01.

### The gut microbiota regulates the expression of the lncRNA-CGB, and such gut commensal-modulated lncRNA involves in active TB infection

**Figure 2.**
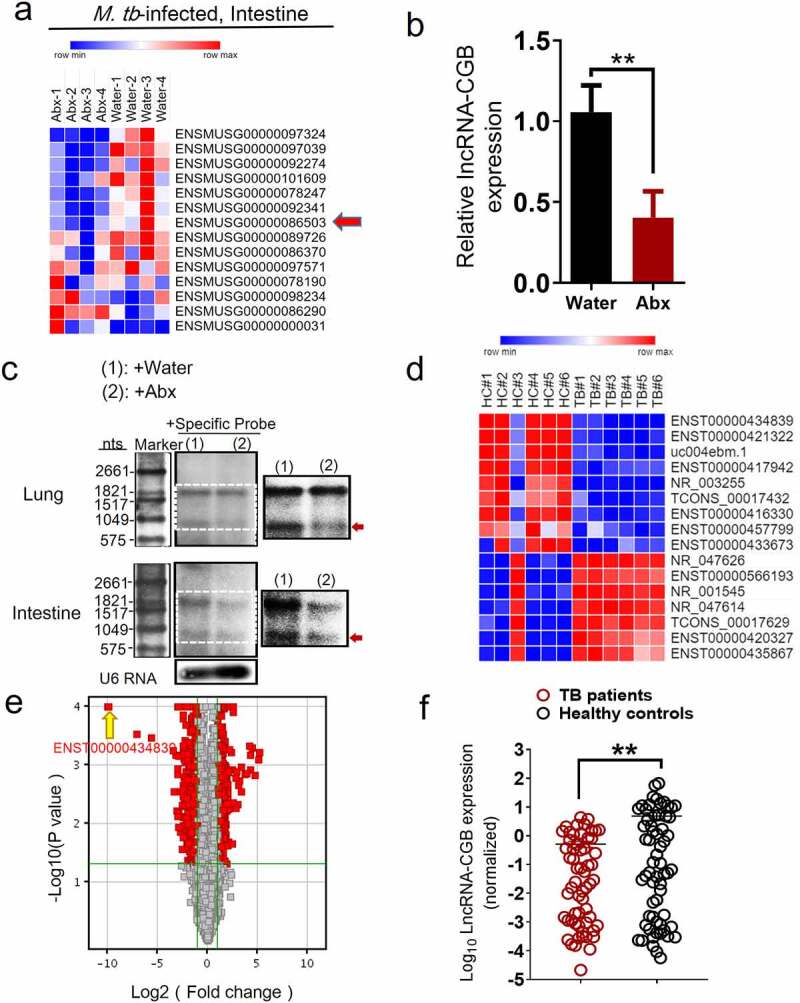
LncRNA-CGB expression was regulated by gut microbiota and involved in TB infection.

To systematically identify gut microbiota-regulated lncRNAs, we then determined whether alteration of gut commensal bacteria would modulate lncRNA expression profiles *via* destruction/aberration of gut microbiota by antibiotic feedings during *M. tuberculosis* infection. RNA sequencing-based transcriptomic analysis was performed to comparatively measure lncRNAs in gut tissues from antibiotics-fed mice and controls during *M. tuberculosis* infection. ~7592 lncRNAs were deregulated in the gut tissues of antibiotics-fed mice during TB infection and among them, lncRNA-ENSMUSG00000086503 was one of the most significantly down-regulated lncRNA signatures when gut microbiota was depleted (Figure 2a). We therefore temporarily named it as lncRNA-CGB (commensal gut bacteria-associated lncRNA) and focused on this lncRNA-CGB for in-depth functional and mechanistic studies.

(a) Heatmap analysis of unsupervised clustering of differentiated lncRNAs between intestines derived from *M. tuberculosis*-infected mice with drinking broad-spectrum antibiotics (N = 4) or water (N = 4). LncRNA-CGB was one of the most significantly down-regulated lncRNAs in the intestines derived from *M. tuberculosis*-infected mice with drinking antibiotics (Abx). (b) qRT-PCR analysis verified that lncRNA-CGB was significantly down-regulated in the lungs derived from *M. tuberculosis*-infected mice with drinking antibiotics (Abx). (c) Northern blot analysis of lncRNA-CGB expression of purified RNA from intestines and lungs that were derived from *M. tuberculosis*-infected mice with drinking broad-spectrum antibiotics (Abx) or water only. Inset: high-contrast view of the region in the dashed box. (d) Heat map analysis of supervised clustering of differentiated human lncRNAs between PBMCs derived from active TB patients (TB) (N = 6) and healthy controls (HC) (N = 6). LncRNA-ENST00000434839 was one of the most significantly down-regulated lncRNAs in active TB patients. (e) Volcano plot showing fold changes for lncRNAs differentially expressed between active TB patients (N = 6) and healthy controls (N = 6). LncRNA-ENST00000434839 was enriched in healthy controls and marked with the yellow arrow. (f) qPCR analysis of lncRNA expression in active TB patients (N = 50) and healthy controls (N = 43). Error bars indicate average values ± SEM. N = 6 SPF mice for each group of mice for each experiment, and results are representative of two independent experiments. Error bars indicated average values ± SEM, **p < .01.

Localization and protein-coding capability analyses of lncRNA-CGB suggested that lncRNA-CGB was located on chromosome X, predominantly localized in the nuclear fractions, was highly conserved in different species including *Homo sapiens* and *Mus musculus* and did not show detectable coding ability or translation events (Supplementary Figure S2a-f).Moreover, qRT-PCR – and northern blot-based quantitative assays demonstrated that antibiotics feeding of *M. tuberculosis*-infected mice while destroying microbiota, significantly reduced expression levels of lncRNA-CGB in lungs and intestines (Figure 2b, c and Supplementary Figure S3). These data implicated that lncRNA-CGB might indeed somehow be involved in regulating TB pathogenesis induced by destruction/aberration of gut microbiota.

To further investigate whether lncRNAs were involved in the gut-lung immune axis in response to *M. tuberculosis* infection, we then evaluated whether TB infections were associated with any gut microbiota-regulated lncRNA signatures using human lncRNAs microarray and hierarchical clustering analyses. The comparative analysis between active TB patients and healthy controls (HC) allowed us to display distinct lncRNAs expression profiles during TB infection. The supervised hierarchical clustering segregation analysis then identified dominant groups of lncRNAs differentially expressed in TB patients (Figure 2d). From 15,683 denoted lncRNAs, 13 lncRNAs were identified to be differentially expressed (TB/HC > 2 or TB/HC< 0.5) in peripheral blood mononuclear cells (PBMCs) between TB and healthy subjects (Figure 2e). Remarkably, among them, lncRNA-ENST00000434839, the human homologue of lncRNA-CGB (Supplementary Figure S2a), was identified as one of the three mostly down-regulated lncRNAs in patients with active TB disease, displaying mean 4.7-fold down-regulation in differentially expressed lncRNAs. High consistency of qRT-PCR validation of lncRNA-ENST00000434839 in a larger cohort of TB patients (N = 50) and healthy controls (HC) (N = 43) further supported that gut microbiota-regulated lncRNA-CGB might somehow involve in active TB disease progression (Figure 2f). To further identify whether this specific lncRNA-CGB was associated with active TB infection, we then explored the expression of lncRNA-CGB in PBMCs derived from *M. tuberculosis*-infected mice by qPCR. Significantly decreased expression of lncRNA-CGB was detected in *M. tuberculosis*-infected mice when compared with those in mock-infected mice (Supplementary Figure S4).

These findings strongly suggested alteration of lncRNA-CGB expression might indeed somehow be involved in immunomodulating gut-lung crosstalk during *M. tuberculosis* infection.

### LncRNA-CGB is required for host resistance against M. tuberculosis infection

**Figure 3.**
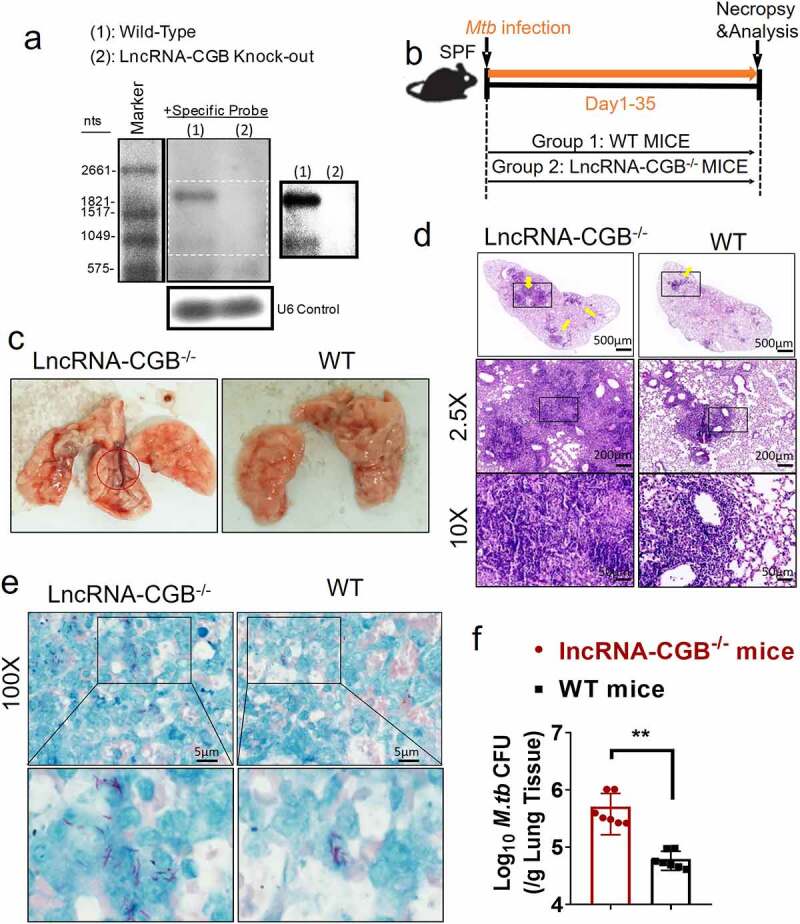
LncRNA-CGB knock-out (KO) mice developed more severe TB than did control mice.

Since gut microbiota was required for developing resistance to TB, and depressed lncRNA-CGB expression patterns correlated with bad outcomes of *M. tuberculosis* infection in mouse model and active TB disease in humans, we then determined the exact role of lncRNA-CGB in *M. tuberculosis* infection. We developed a lncRNA-CGB genomic knock-out (KO) mouse strain using the CRISPR-Cas9 system deletion of the lncRNA regions (Supplementary Figure S5a). Genomic deletion and loss of lncRNA-CGB transcripts in the lymphocytes from the lncRNA knock-out (KO) mice were confirmed by northern blotting (Figure 3a) and PCR analysis (Supplementary Figure S5b). The genomic knock-out (KO) did not interfere with the expression of flanking genes, and there was a lack of any abnormal findings for the lncRNA knock-out (KO) mice. We then examined whether *in vivo* deficiency of lncRNA-CGB would result in a beneficial or detrimental effect on *M. tuberculosis* infection (Figure 3b). Importantly, lncRNA-CGB knock-out (KO) mice developed much more severe TB disease after *M. tuberculosis* infection than did wild-type animals. Virtually, gross pathology and hematoxylin and eosin (H&E) staining of lung sections revealed that lncRNA-CGB knock-out (KO) mice developed more severe disruption of lung structure and larger scale granuloma lections than did control mice (Figure 3c, d). In addition, higher bacillus burdens in lungs derived from lncRNA-CGB knock-out (KO) mice were observed than did control mice (Figure 3e, f).

(a) Northern blotting analysis of lncRNA expression in splenocytes isolated from wild-type (WT) or lncRNA-CGB KO mice. (b) The experimental diagram showed that WT or lncRNA-CGB KO mice were infected with *M. tuberculosis* and sacrificed at the indicated time point. (c) Gross pathology of representative lungs isolated from WT and lncRNA-CGB KO mice. The red circle marked more severe pathological lesions. (d) H&E staining of sections of representative lungs derived from *M. tuberculosis*-infected lncRNA-CGB KO or WT mice. Yellow arrows heads marked the granuloma lesions or accumulation of inflammatory cells in the lungs. Note that larger-scale and more granuloma lesions were observed in lncRNA-CGB KO mice. (e) Macroscopic evaluation of *M. tuberculosis* bacilli burdens and distribution on the sections of lungs isolated from *M. tuberculosis*-infected WT and lncRNA-CGB KO mice. Representative fields were shown. Note that more bacilli positive for acid-fast staining was observed in lesions of *M. tuberculosis*-infected lncRNA-CGB KO mice. Lower panel: higher-resolution view of the regions as indicated by the box in the upper panel. (f) *M. tuberculosis* CFU analysis of bacillus burdens in the lungs (per gram) of *M. tuberculosis*-infected WT or lncRNA-CGB KO mice. N = 7 SPF mice for each group for each experiment with at least two biological repeats. Error bars indicate average values ± SEM, **p < .01.

To confirm whether human lncRNA-CGB also played a role in mediating anti-TB resistance as mouse lncRNA-CGB did, we developed a human T cell adoptive transfer-based *M. tuberculosis* infection therapeutics study model with lncRNA-CGB knock-down in CD3 + T cells, as we previously did.^28^ Briefly, CD3 + T cells and CD14+ monocytes purified from PBMCs of active TB patients were firstly transduced with lentiviral (LV) vector encoding shRNA targeting lncRNA-CGB or LV vector only (LV-Ctrl). LV-lncRNA–transduced (lncRNA-CGB–depressed) and LV-Ctrl–transduced CD3 + T cells were then transferred into each of recipient *M. tuberculosis*-infected mice which had also received autologous monocytes from TB patients to facilitate *M. tuberculosis* infection. We found that lncRNA-CGB knock-down impaired the ability of human CD3 + T cells to limit *M. tuberculosis* replications and infections in this T cell adoptive transfer-based *M. tuberculosis* infection therapeutics study model (Supplementary Figure S6a, b). Thus, these results suggested that both mouse and human lncRNA-CGB played a critical role in limiting *M. tuberculosis* infection and TB pathology.

Taken together, we found that gut bacteria might mediate host resistance against TB through sustaining or enhancing the expression of lncRNA-CGB.

### B. fragilis, a significantly altered microbiota during TB, is a key mediator of the lncRNA-CGB expression profile

**Figure 4.**
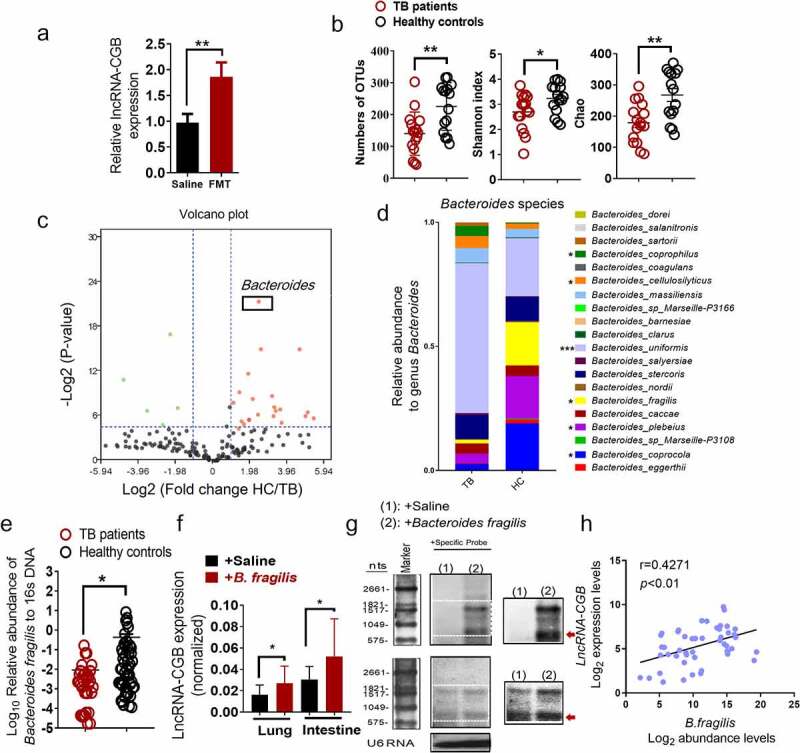
*B. fragilis* was identified as a significantly altered gut microbiota to orchestrate the expression of lncRNA-CGB during TB infection.

We then identified which component of gut microbiota mediated the expression and function of lncRNA-CGB. We firstly colonized antibiotic-treated mice with microbiota from healthy participants and then measured the expression of lncRNA expression in the lungs. Fecal microbiota restoration substantially increased lncRNA-CGB expression in lung tissues (Figure 4a). This indicated that human gut microbiota indeed could modulate lncRNA-CGB expression.

(a) LncRNA-CGB expression of representative lungs derived from the *M. tuberculosis-*infected mice with the treatment of saline or FMTs of gut microbiota from healthy individuals. (b) The Numbers of OTUs, Shannon Wiener index, Chao index showing alpha-diversity are displayed between active TB patients (TB) (N = 16) and healthy controls (HC) (N = 15). Significantly lower species richness and diversity in TB patients’ samples (TB) were observed when compared to healthy controls (HC). (c) Volcano plot of differential bacterial abundance analysis at genus level between active TB patients and healthy controls. *Bacteroides* was enriched in healthy controls. (d) Relative abundance of *Bacteroides* species (percentage of the total genus *Bacteroides*) in healthy controls and active TB patients. (e) qPCR analyses *B. fragilis* abundance in feces derived from TB patients (N = 50) or healthy controls (HC) (N = 43). Much lower enrichment of *B. fragilis* was found in the feces from a large cohort of TB patients. (f-g) *B. fragilis*-regulated anti-TB immunity coincided with an increased expression of lncRNA-CGB in SPF mice receiving *B. fragilis* transfer/colonization. The qRT-PCR analysis and northern blot analysis (inset: high-contrast view of the region in the dashed box) of lncRNA-CGB expression in intestines and lungs derived from *M. tuberculosis*-infected mice orally administrated with *B. fragilis*(1 × 10^9^) and saline control. (h) Correlation analysis between *B. fragilis* abundance and lncRNA-CGB expression in TB patients. N = 6 SPF mice for each group of mice for each experiment, and results are representative of three independent experiments. Error bars indicated average values ± SEM, *p < .05, **p < .01.

We therefore performed a detailed comparison of gut microbial profiles using 16S rRNA gene sequencing in fecal samples from active TB patients and healthy controls (HC). The baseline characteristics of the participants are shown in Table 1. Consistent with previous reports,^30–32^ we observed significantly lower species richness in TB patients’ samples (TB) compared to healthy controls (HC) (Wilcoxon rank-sum test; Figure 4b). Consistently, the Shannon index and the Chao1 Index, indicating within-sample diversity, were much lower for the TB patient group than that for the healthy controls (HC) (*p-*values<0.01, <0.01; Wilcoxon rank-sum test; Figure 4b). These data suggested that microbial diversity was decreased in active TB patients and *M. tuberculosis* infection exhibited alterations of the discriminatory genus of gut microbiota.

**Table 1.** Demographic and clinical characteristics of active TB patients (TB) and healthy controls (HC)

	Healthy controls (HC)	TB patients (TB)
Sample size	43	55
Female	28 (65.1%)	29 (52.7%)
Male	15 (34.9%)	26 (47.3%)
Age (median [interquartile range])	27 (24–35)	34 (26–40)
Smear grade None 1+ 2+ 3+ 4+	43 N/A N/A N/A N/A	7 (12.7%) 18 (32.7%) 16 (29.1%) 9 (16.4%) 5 (9.1%)
Chest X-ray Nodules and fibrotic scars Infiltrates or consolidations Cavity Atelectasis Pleural effusion Bronchiectasis	N/A N/A N/A N/A N/A N/A	40 (72.7%) 44 (88.0%) 26 (47.3%) 16 (29.1%) 6 (10.9%) 14 (25.5%)
Antibiotics treatment	N/A	None

To analyze the gut microbial profiles of the study participants and identify the exact gut bacteria driving lncRNA-CGB alteration, a comprehensive comparison between the patients with TB and controls in terms of the abundance of genera was performed. Of the 164 genera identified, 28 (17.1%) were found to have significant differential log abundances between both groups (Figure 4c). Specifically, we found that the relative abundance of the genus *Bacteroides* was visibly lower in TB patients compared with controls (Figure 4c). Considering that previous reports have shown a lower abundance of the genus *Bacteroides* in children with pulmonary tuberculosis^33^ and *Bacteroides* has been implicated to play a critical role in mediating immune resistance against a variety of diseases such as cancers,^34,35^ we then focused on the *Bacteroides* species to investigate whether the altered abundance of *Bacteroides* species was associated with the development and progression of human TB infection. Notably, *B. fragilis* was identified as one of the most significantly differently and abundantly expressed species within the genus *Bacteroides* in 16S rRNA gene sequencing analysis, displaying mean 12.13-fold up-regulation in 20 species of gut microbiota from healthy controls over TB patients (Figure 4d). Furthermore, in order to verify the validity of 16S rDNA analysis, qRT-PCR analysis targeting *B. fragilis* was performed in a larger cohort of TB patients (TB) (N = 50) and healthy controls (HC) (N = 43). qRT-PCR analysis showed a much lower enrichment of *B. fragilis* in the feces from a large cohort of TB patients (Figure 4e).

To further validate the intestinal microbiota changes were due to *M. tuberculosis* infection, we next comparatively the compositions of the microbiome in *M. tuberculosis-*infected or uninfected mice by qPCR. In agreement with previous study,^36^ we observed similarly decreased trends of gut microbiota diversity during murine *M. tuberculosis* infection. Interestingly, the genus that showed higher abundance in the feces of healthy controls (HC) also displayed higher abundance levels in uninfected mice, including genus *Veillonella, Blautia, Neisseria Fusobacterium, Desulfovibrio, Bacteroides, Lactobacillus, Enterococcus* and *Campylobacter* (Supplementary Figure S7). More importantly, the relative abundance of genus *Bacteroides* was significantly decreased in the *M. tuberculosis*-infected mice, which is consistent with those alterations in TB patients’ gut microbiome (Supplementary Figure S7). Moreover, such alterations of gut microbiome in response to *M. tuberculosis* infection did not induce observable histopathological changes of cecum in Hematoxylin and Eosin (H&E) staining (Supplementary Figure S8).

Thus, these data further suggested that *B. fragilis* was a gut microbial taxa that was the most significantly altered by active TB infection.

### LncRNA-CGB is upregulated by B. fragilis and is clinicopathologically related to TB features and outcomes

Given the appreciated importance of *B. fragilis* in governing microbiota-host interaction,^37^ shaping host immune response^34,37,38^ and regulating T cell immunity,^39^ we further hypothesized that *B. fragilis* might play, or be at least one of, the most important roles in regulating lncRNA-CGB expression and gut-lung immune axis, which therefore determined the outcomes of *M. tuberculosis* infection. To test this hypothesis, we quantified the lncRNA-CGB expression after oral administration of nontoxic commensal gut bacteria, *B. fragilis*. Remarkably, analyses based on qRT-PCR and northern blotting revealed that *B. fragilis* treatment increased or sustained the lncRNA-CGB expression in *M. tuberculosis*-infected mice (Figure 4f, g). In consistent with mouse data showing the positive relationship between higher lncRNA-CGB expression and *B. fragilis* abundance, we also observed a significant correlation between higher abundance of *B. fragilis* in fecal samples and the lncRNA-CGB up-regulation in active TB patients (Figure 4h). Sputum smear positivity in patients with active TB are usually correlated with higher disease severity.^40^ Thus, to provide further evidence showing the association between the relative abundance of *B. fragilis* and TB infection, we further examined the relationship between the levels of *B. fragilis* abundance in fecal samples and the grades of smear positivity. A trend toward increased levels of the *B. fragilis* with decreasing numbers of *M. tuberculosis* bacteria in the sputum was observed (Supplementary Figure S9). A significant negative correlation between the relative abundance of *B. fragilis* and sputum smear grades was found in smear-positive TB patients (r = −0.9531, *p* < .05) (Supplementary Figure S9).

Therefore, these data from mouse model and humans collectively indicated that lncRNA-CGB expression was dictated by gut bacteria, and such gut commensal-modulated, variable lncRNA-CGB expression patterns might be a mechanism for a selected gut commensal governing gut-lung immune interaction axis in response to *M. tuberculosis* infection.

### *B.* *fragilis* enhanced host resistance against M. tuberculosis infection in mice

**Figure 5.**
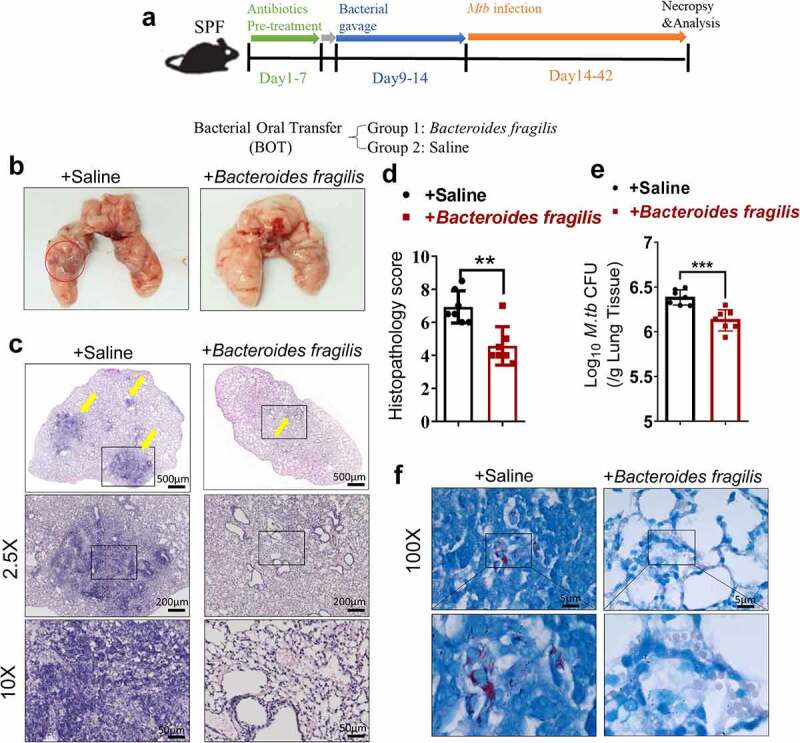
Direct oral administration of *B. fragilis* protected mice against *M. tuberculosis* infection.

LncRNA-CGB, which was dictated by *B. fragilis*, conferred the beneficial effects on host resistance to *M. tuberculosis* infection, raised a critical question as to whether dominant or abundant bacteria *B. fragilis* was responsible for conferring anti-TB beneficial effects. To define the role of *B. fragilis* during *M. tuberculosis* infection, we assessed *B. fragilis* in oral transfer for its ability to function as an active anti-TB component in TB-associated or TB-altered gut bacteria (Figure 5a). Mice that received bacteria oral transfer (BOT) of *B. fragilis* displayed significantly attenuated tissue pathology and decreased *M. tuberculosis* burdens while saline-treated mice showed more severe lung pathology characterized by large-scale damage of lung structure and defined granuloma (Figure 5b-d). Moreover, *B. fragilis*-treated mice showed much lower *M. tuberculosis* CFU numbers in lungs than that of saline-treated mice (Figure 5e), and lung sections derived from *B. fragilis*-treated mice contain fewer acid-fast-staining-positive *M. tuberculosis* bacilli (Figure 5f). Interestingly, the structure of cecum exhibited no significant differences on *M. tuberculosis*-infected lncRNA-CGB knock-out/wild type mice with/without oral treatments of *B. fragilis* and their matched controls (Supplementary Figure S8). Taken together, *B. fragilis* acted as a beneficial regulator in conferring immune control of pulmonary *M. tuberculosis* infection and TB pathology.

(a) Experimental diagram to test the role of selective commensal gut bacteria in control of *M. tuberculosis* infection and TB pathology. Antibiotics pre-treated mice were orally administrated with *B. fragilis* (1 × 10^9^) or saline every three days for three total treatments followed by aerosol *M. tuberculosis* infection. (b) Gross pathology of representative lungs derived from the *M. tuberculosis*-infected mice with indicated *B. fragilis* or saline treatment. The red circle marked the damages, necrosis, and severe hemorrhage in the lungs. (c) H&E staining of representative lungs derived from the *M. tuberculosis*-infected mice with indicated *B. fragilis* or saline treatment. Yellow arrows marked the lung pathology of granuloma and infiltration of inflammatory cells. Note that *M. tuberculosis*-infected mice with BOT of *B. fragilis* showed much milder TB lung pathology. (d) Quantitative representation of the lung histopathology in mice with indicated BOT or saline treatment after infection with *M. tuberculosis*. The blinded histopathology score was averaged for seven mice per experimental group per time point. (e) *M. tuberculosis* burdens determined by CFU quantitative analyses in the lungs (per gram) in *M. tuberculosis*-infected mice with indicated BOT or saline treatment. (f) Macroscopic evaluation of *M. tuberculosis* bacilli numbers on the sections of lungs isolated from the lungs of *M. tuberculosis*-infected mice with indicated BOT or saline treatment by acid-fast staining. Note that appreciably fewer bacilli positive for acid-fast staining were observed in the lungs of *M. tuberculosis*-infected mice with oral gavage of *B. fragilis*. Lower panel: higher-resolution view of the regions in the box. Representative fields were shown. N = 7 mice for each group of mice for each experiment with at least two biological repeats. Error bars indicate average values ± SEM, **p < .01, ***p < .001.

### LncRNA-CGB mediates gut microbiota-reduced host susceptibility to TB by inducing IFN-γ production

**Figure 6.**
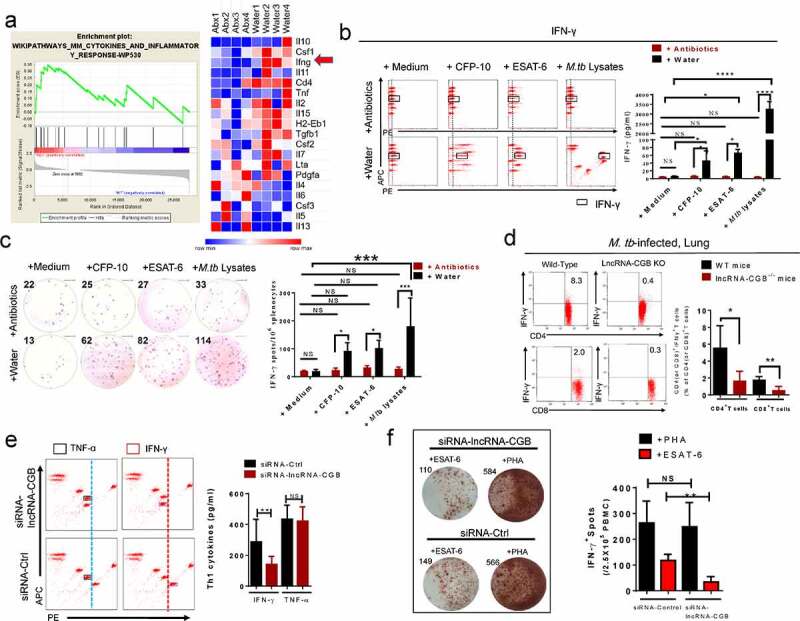
Gut microbiota and its regulated lncRNA-CGB modulate *de novo* IFN-γ responses during *M. tuberculosis* infection.

We then examined how commensal bacteria was involved in mediating protective immunity against *M. tuberculosis* infection. The gene set enrichment analysis (GSEA) of the lungs from antibiotics-treated or control mice (GSEA false discovery rate [FDR] respectively q < 0.05; Figure 6a) suggested that gut microbiota might modulate cytokines and inflammatory responses during *M. tuberculosis* infection. Furthermore, RNA sequencing-based transcriptomic analysis also demonstrated significantly down-regulated IFN-γ transcriptional responses in the lungs of antibiotics-fed mice during *M. tuberculosis* infection (Figure 6a). It is noteworthy that IFN-γ is absolutely required for immune resistance to TB,^41^ and gut microbiota is critically important for the production of IFN-γ.^20,42^ We therefore postulated that IFN-γ might be a key mediator linking gut microbiota, lncRNA profiles and pulmonary immune homeostasis.

(a) GSEA of signaling pathway targets in samples from antibiotics-feeding*M. tuberculosis*-infected mice versus samples from *M. tuberculosis*-infected mice with drinking water. Relative abundance of inflammatory and cytokine pathway target genes in lungs derived from *M. tuberculosis-*infected mice with drinking broad-spectrum antibiotics (N = 4) or water only (N = 4). Inflammatory and cytokine pathway target genes derived from the GSEA leading-edge, set #1. Note that IFN-γ (*ifng*) was one of the most significantly down-regulated genes in the lungs derived from *M. tuberculosis*-infected mice with drinking antibiotics. (b) Representative CBA/flow cytometric fluorescent clustering and bar graphic analysis of IFN-γ expression in culture supernatants of lymphocytes isolated from lungs of *M. tuberculosis*-infected mice with drinking broad-spectrum antibiotics or water only. Lymphocytes isolated from the lungs of *M. tuberculosis*-infected mice were cultured (1 × 10^6^/culture well) in the presence or absence of *M. tuberculosis* lysates (5 μg/ml) for 3 days and subjected to CBA/flow cytometric analysis of IFN-γ expression. Fluorescent clusters representing IFN-γ expression were marked with black squares and the relative IFN-γ concentration was marked with a shift of dashed lines. (c) Representative ELISPOT spot images and bar graphic analysis of numbers of IFN-γ-expressing lymphocytes derived from lungs of *M. tuberculosis*-infected mice with drinking antibiotics or water only. Lymphocytes isolated from lungs were cultured in the presence or absence of *M. tuberculosis*-specific CFP-10 (5 μg/ml) peptides, ESAT-6 peptides (5 μg/ml) or whole *M. tuberculosis* lysates (5 μg/ml) for 48 hours before subjecting to IFN-γ-ELISPOT analysis to determine the numbers of IFN-γ-expressing lymphocytes. (d) Representative flow cytometric dot plots and bar graphic analysis of de novo IFN-γ expression in CD4+ and CD8 + T cells in the lungs derived from *M. tuberculosis*-infected lncRNA-CGB KO and wild-type (WT) mice. Note that lung homogenates were directly subjected to ICS/Flow cytometric analysis to analyze the IFN-γ expression without ex vivo antigenic or anti-CD3/anti-CD28 re-stimulation.(e) Representative CBA/flow cytometric fluorescent clustering and bar graphic analysis of IFN-γ expression in culture supernatants of CD3 + T cells isolated from active TB patients (N = 9) with transfection of siRNA targeting lncRNA-CGB (siRNA-lncRNA-CGB) or control siRNA (siRNA-Ctrl) for 3 days and subjected to CBA/flow cytometric analysis of IFN-γ expression. (f) Representative ELISPOT spot images and bar graphic (n = 6 patients) analysis of numbers of IFN-γ-expressing cells derived from active TB patients. PBMCs (1 × 10^6^) isolated from active TB patients were transfected with siRNA targeting lncRNA-CGB (siRNA-lncRNA – CGB) or control siRNA (siRNA-Ctrl) for 72 hours and cultured in the presence or absence of *M. tuberculosis*-specific ESAT-6 peptides (5 μg/ml) or PHA (Phytohemagglutinin) for 48 hours before subjecting to IFN-γ-ELISPOT analysis to determine numbers of IFN-γ – expressing cells. N = 6 SPF mice for each group of mice for each experiment and results are representative of three independent experiments. Error bars indicated average values ± SEM, *p < .05, **p < .01, ***p < .001, ****p < .0001, NS: no statistical significance.

Concurrently, antibiotics feedings of *M. tuberculosis*-infected mice shut down or remarkably reduced the ability of lung lymphocytes to mount *M. tuberculosis*-specific IFN-γ responses as revealed by flow-cytometry-based quantitation (Figure 6b) and IFN-γ-ELISPOT assays (Figure 6c). Moreover, antibiotics feedings significantly decreased *de novo* production of IFN-γ by intestinal CD4+ and CD8 + T cells derived from *M. tuberculosis*-infected mice without further *ex vivo* re-stimulation with *M. tuberculosis* antigens (Supplementary Figure S10).

In addition, lncRNA-CGB knock-out indeed reduced IFN-γ expression by CD4+ and CD8 + T cells during *M. tuberculosis* infection in mice (Figure 6d), implicating that commensal bacteria-dictated lncRNA-CGB was involved in mediating IFN-γ expression and therefore conferred beneficial effect against *M. tuberculosis* infection. In agreement with these mouse data, knock-down of lncRNA-CGB in CD3 +^ ^T cells derived from active TB patients also led to an inhibited expression of IFN-γ (Figure 6e, f). In parallel, oral administration of *B. fragilis* allowed increased induction of IFN-γ expression during *M. tuberculosis* infection *in vivo and ex vivo* (Supplementary Figure S11). These results suggested that IFN-γ was one of the key immune mediators linking gut-lncRNA-CGB-lung axis.

Taken together, the above findings allowed us to hypothesize a potential biological sequence or pathway: gut microbiota →lncRNA-CGB→IFN-γ expression →resistance to TB.

### LncRNA-CGB directly interacted with EZH2 and negatively regulated H3K27Me3, leading to increased TB-specific IFN-γ expression and immunity against M. tuberculosis infection

**Figure 7.**
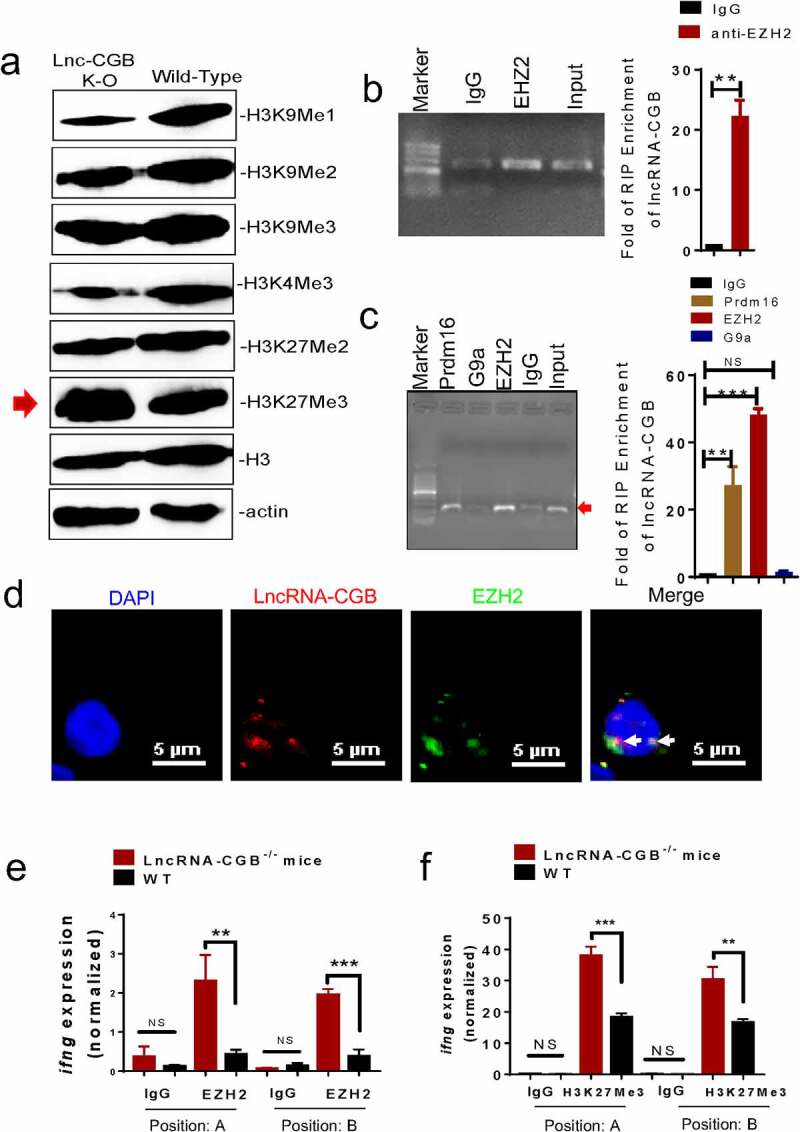
LncRNA-CGB negatively regulates H3K27Me3 in IFN-γ (*ifng*) promoter *via* physically interacting with EZH2.

We next sought to investigate the molecular mechanisms by which antibiotics’ destruction of microbiota depressed lncRNA-CGB and IFN-γ responses leading to a loss of immune resistance to *M. tuberculosis* infection. Given that emerging evidence has highlighted the critical roles for lncRNAs in mediating immune response genes^43^ or T cell immune responses^28,44–47^ by directly or indirectly interacting with epigenetic modifiers,^28,44,47–55^ we then performed global histone methylation analyses in lymphocytes isolated from the lungs of *M. tuberculosis*-infected lncRNA-CGB knock-out (KO) mice or wild-type mice. We found that genomic knock-out of lncRNA-CGB increased expression of H3K27Me3 but reduced H3K9Me1 and H3K4Me3 (Figure 7a), implicating that lncRNA-CGB knock-out (KO) led to a globally more repressive chromatin state during *M. tuberculosis* infection. Since tri-methylation (Me3) at H3K27 could be catalyzed by the EZH2 subunit of histone methyltransferase Polycomb Repressive Complex 2 (PRC2),^56^ we therefore presumed that lncRNA-CGB might directly interact with EZH2/H3K27Me3 epigenetic programming of IFN-γ transcription. To test this, we first predicted the interaction potential between lncRNA-CGB and EZH2. We identified a 51-nt motif with a predicted secondary structure in lncRNA-CGB similar to a previously validated 89-mer EZH2 interacting motif in lncRNA-*Hotair*^56^ (Supplementary Figure S12a), but the interaction potential of EZH2/lncRNA-CGB appeared to be stronger than (or at least as strong as) that of EZH2/lncRNA-*Hotair* (Supplementary Figure S12b,c).

(a) Representative Western blotting analyses of H3K9Me1, H3K9Me2, H3K9Me3, H3K4Me3, H3K27Me2, and H3K27Me3 in lymphocytes isolated from lungs of *M. tuberculosis*-infected lncRNA-CGB KO and wild-type (WT) mice. (b) Gel electrophoresis (left) and qRT-PCR (right) analysis of lncRNA-CGB retrieve in immunoprecipitation (IP) by IgG, anti-EZH2, anti-PRDM16, or anti-G9a antibody in lysates of T cells isolated from PBMC of active TB patients. The levels of qRT-PCR products were expressed as a percentage of input RNA. (c) Gel electrophoresis (left) and qRT-PCR (right) analysis of lncRNA-CGB retrieve in immunoprecipitation (IP) by IgG and anti-EZH2 antibody in lysates of lymphocytes isolated from lungs of *M. tuberculosis*-infected mice. The levels of qRT-PCR products were expressed as a percentage of input RNA. (d) Confocal microscopic images of RNA FISH assay of lncRNA-CGB and immunofluorescence analysis of EZH2 show that EZH2 co-localizes with lncRNA – CGB in the nucleus of T cells isolated from patients with active TB. More than 20 cells were examined and had similar results. White arrowheads marked the EZH2/lncRNA-CGB co-localization.(e) ChIP-qPCR analysis, using anti-EZH2 and IgG Control antibody, at positions A and B of promoter regions of IFN-γ (*ifng*) in lymphocytes isolated from lungs of *M. tuberculosis*-infected lncRNA-CGB KO and wild-type (WT) mice. (f) ChIP-qPCR analysis, using anti-H3K27Me3 and IgG Control antibody, at positions A and B of promoter regions of IFN-γ (*ifng*) in lymphocytes isolated from lungs of *M. tuberculosis*-infected lncRNA-CGB KO and wild-type (WT) mice. N = 6 SPF mice for each group of mice for each experiment, and results are representative of three independent experiments. Error bars indicated average values ± SEM, **p < .01, ***p < .001, NS: no statistical significance.

We then performed RNA immunoprecipitation (RIP) retreat assay using CD3 + T cells isolated from active TB patients. Indeed, we found that lncRNA-CGB was immunoprecipitated with both EZH2 and Prdm16 in lysates from CD3 + T cells of TB patients, with no or very little in G9a or IgG controls (Figure 7b). Similar amounts of lncRNA-CGB/EZH2 interactions were also found in lymphocytes derived from the lungs of *M. tuberculosis*-infected mice (Figure 7c). Consistently, confocal microscope-based fluorescence *in situ* hybridization (FISH) analysis of lncRNA-CGB and immunofluorescent staining of EZH2 showed that significant amounts of EZH2 co-localized with lncRNA-CGB in the nucleus of CD3 + T cells from patients with active TB (Figure 7d). Concurrently, weak interaction events between lncRNA-CGB and EZH2 were observed in confocal imaging and RIP in PBMCs derived from healthy controls (Supplementary Figure S13), further supporting the view that lncRNA-CGB interacted with the EZH2 subunit of histone methyltransferase PRC2 and thereafter regulated EZH2 and H3K27Me3 epigenetic programming in the *ifng* promoter. Besides, ChIP-qPCR analysis showed that lncRNA-CGB knock-out increased EZH2 abundance and enhanced tri-methylation (Me3) of H3K27 in the *ifng* promoter during *M. tuberculosis* infection (Figure 7e, f), suggesting that lncRNA-CGB was required to inhibit EZH2 translocation to the *ifng* promoter where they might mediate H3K27Me3 for inhibition of IFN-γ expression.

The studies presented here identified that lncRNA-CGB governed by *B. fragilis* and lncRNA-dictated IFN-γ expression were key mediators of gut-lung axis (Supplementary Figure S14). Thus, this work may shed new light on developing a microbiota-based treatment against lung diseases such as *M. tuberculosis* infection.

## Discussion

While an emerging area of intense interest is the role of gut-lung axis in the pathogenesis of lung diseases,^2,57^ accumulating evidence has demonstrated that gut microbiota plays a critical role in mediating gut-lung axis.^58,59^ However, due to the complex crosstalk, the exact mechanisms through which the gut impacts lung health or disease and vice versa are only starting to be uncovered. The gut-lung axis allows for the passage of endotoxins, microbial metabolites, cytokines, or hormones into the bloodstream connecting the gut niche with that one of the lungs. However, mechanistic insights into the pathways and mediators involved in gut-lung axis are largely unexplored. For the first time, this work has characterized the previously unrecognized importance of a commensal gut bacteria-modulated lncRNA signature in mediating host defense mechanisms against deadly lung diseases, such as TB, *via* epigenetic programming of expression of an immune mediator.

Although it has been firmly established that the intestinal microbiota may exert a tremendous impact on the development and homeostasis of the immune system locally and systemically, our understanding of the exact mechanisms by which lung health is determined by intestinal microbiota is still at the beginning. While signals delivered by gut microbes were documented to influence the functions of innate immune cells,^4,18,60^ adaptive T cells, such as CD4+ TCRαβ T helper (Th) cells, were also influenced by the intestinal epithelium-associated microbiota.^61^ Pulmonary TB disease is a classic example of how a successful control of *M. tuberculosis* infection relies on delicate orchestration between adequate immune responses and inhibited activation and functional exertion of inflammatory networks.^62,63^ However, there is limited evidence regarding potential mechanistic pathways on gut-lung interactions that may orchestrate immune balance in both gut-lung axis that may further affect TB susceptibility, manifestation and progression. Our results provide the first line of evidence suggesting that the commensal gut bacteria-related lncRNA-CGB is linked to the regulation of the gut-lung axis for protective immunity against lung pathogenesis.

This work highlighted the importance of lncRNAs in shaping and promoting IFN-γ expression and function to support pulmonary homeostasis. The IFN-γ-deficient mice displayed significantly higher pulmonary *M. tuberculosis* burdens,^41^ and regulation events from a gut microbiota-associated lncRNA in considerable responses by CD4 + T and CD8 + T effector subpopulations in naïve settings were observed in this work. Thus, it is not surprising to see changes in IFN-γ-driven anti-TB immunity in lncRNA-CGB knock-out mice. Also, relatively smaller alterations in *M. tuberculosis* CFU numbers were observed in lncRNA-CGB knock-out mice in this study than that of *ifng* knock-out mice.^41^ This difference may be explained by the notion that lncRNA-CGB-enhanced anti-TB immunity may be partially compromised by other gut bacteria-regulated lncRNAs which are also involved in the immune network regulation during *M. tuberculosis* infection. Furthermore, since increased IFN-γ productions may lead to over-reacted immune response and immunopathology and significant fractions of *M. tuberculosis*-infected individuals with active TB disease express quite appreciable amounts of IFN-γ, enhanced expression of IFN-γ driven by lncRNA-CGB may not mean full immune protection against *M. tuberculosis* infection.^64^ Thus, full investigations of more comprehensive lncRNAs-gut microbiota-immune network should be required for uncovering exact TB pathogenesis mechanisms.

Although it was anticipated that TB can alter abundance, composition and diversity in gut microbiota, exact TB-induced alterations of gut microbial components and how such TB-altered gut microbial species orchestrate pulmonary or systemic immunity against TB infection are unclear. We have identified that *B. fragilis* was a most significantly altered gut microbiota taxa that discriminated active TB patients from healthy controls. A recent study has reported that the relative abundance of *B. fragilis* might be increased after anti-TB treatment,^65^ suggesting the recovery in the functional ability of *B. fragilis* after anti-TB treatment. The importance of *B. fragilis* in mediating immune protection or homeostasis against TB further gained support from our study wherein oral administration of *B. fragilis* conferred homeostatic benefits and immune resistance against *M. tuberculosis* infection. Since the structure and function of gut microbiota are dynamically regulated by genetics, environment, and food, the pre-treatment of antibiotics may help the mice orally treated with human gut microbiota or a single strain bacteria tend to be at the same baseline in terms of structure and function of their gut microbiota in our study. However, the true impacts and underlying mechanisms of the baseline of gut microbiome on treatments of FMT or gut microbiota interference during TB infection need further study.

Moreover, our study revealed that a significant shift of gut bacteria toward beneficial microbial consortium could be highly associated with the sustaining of antibiotics-depressed lncRNA-CGB expression and production of protective IFN-γ response against TB by *B. fragilis*. The data therefore consistently point to the notion that lncRNA is a decisive mediator for *B. fragilis* to control TB.

To date, commensal probiotic *B. fragilis* or short chain fatty acids (SCFAs) fermented from *B. fragilis* have been shown to inhibit inflammation and infection by pathogenic bacteria and support cancer therapy *via* microbe-host immune interactions.^34,60^ Other views also suggest that *B. fragilis* may control pathogenesis by readjusting the composition of the gut microbiota or by enhancing the intestinal barrier defense system.^66^ Notably, the roles and mechanisms of *B. fragilis* in the regulation of any lncRNA in the case of TB have not been reported elsewhere. Our current study elucidated that gut microbiota was important for the control of *M. tuberculosis* infection through sustaining expression of lncRNA-CGB and underscored the concept that gut microbiota not only directly mediated immune responses but also regulated non-coding genes to induce the immunomodulatory effects in response to *M. tuberculosis* infections.

While it is still at the beginning to understand exact molecular mechanisms regulating the gut-lung axis, our epigenetic mechanism study reveals that lncRNA-CGB inhibits H3K27Me3 programming in IFN-γ promoter and allows for IFN-γ expression and subsequently protective homeostasis and outcomes during *M. tuberculosis* infection. Notably, lncRNA-CGB binding to EZH2 did not seem to directly repress PRC2 enzymatic activity of EZH2. Rather, such binding probably interferes with EZH2 genomic targeting in the *ifng* promoter through antagonizing activities of EZH2, leading to relieving the suppressive function of EZH2 in the *ifng* promoter. We have observed a reduced expression of H3K9Me1 in *M. tuberculosis-*infected lncRNA-CGB KO mice and revealed interactions between lncRNA-CGB and Prdm16, an H3K9Me1 modifier. It will be interesting to further determine whether lncRNA-CGB action may also require other PRC2-binding lncRNAs or other involved histone modifiers.

As systemic activation *via* translocation of gut microbial products from the intestine into the blood circulation has been well-documented,^2^ we thus postulate that there are several possible scenarios for how gut microbial products may impact the anti-TB immunity: firstly, gut microbial products such as SCFAs may be released into the bloodstream and reach lungs and thereby influencing the expression of lncRNA-CGB in resident T subsets in lungs and triggering them to further exert effector functions and thus restrict intracellular *M. tuberculosis*; secondly, naïve T cells in gut-associated lymphoid tissues may be directly activated by gut microbial products *via* sustaining or enhancing their lncRNA-CGB expression and then migrate into lung tissues, where they facilitate anti-*M. tuberculosis* immune functions; thirdly, gut microbial products may systemically protect T cell viability from entering into exhaustion *via* regulating expressions of inhibitory markers such as TIM-3, PD-1 and maintain their anti-*M. tuberculosis* immune functions in either lung or gut compartments. However, these postulations need to be further investigated. Recent studies suggest that the microbiota and its metabolites influence genomic reprogramming such as DNA methylation and histone modification.^67^ Previous work on the correlation of the gut microbiota dominant group *Bacteroidetes* and differential methylation status of gene promoters in cardiovascular diseases^68^ hint that the control of lncRNA-CGB expression during TB infection evolved as one potential mechanism for impaired anti-TB immunity.

It remains extremely difficult to control TB due to the broad emergence of drug-resistant strains of *M. tuberculosis*, insufficient BCG vaccine efficacy against pulmonary TB and HIV-1 co-infection.^69^ Developing new strategies for effective control of TB, such as better vaccines or new therapeutics, can be facilitated by identifying a new paradigm of protective immunity^62^and uncovering previously unknown pathogenesis mechanisms during *M. tuberculosis* infection.^70,71^ Our gut microbiota approach therefore might potentially serve as adjunctive therapeutics against TB although in-depth studies in animals and humans are required to confirm that lung protections and homeostasis could really be achieved *via* oral administrations of a single-strain gut bacteria or a rationally-designed gut bacteria consortium.

Overall, the current study defines gut microbiota functions and mechanisms for maintaining lung immune homeostasis through modulating lncRNA expression. Gut dysbiosis can dictate expression patterns of lncRNA in both gut and lung and induce immune dysfunction at distant organs. We also made useful observations implicating microbiota-mediated regulatory pathways: gut microbiota →lncRNA-CGB →EZH2/H3K27Me3 epigenetics →IFN-γ expression → anti-TB immunity. Thus, this work not only provides a novel framework and in-depth understanding of significances and underlying mechanisms in homeostatic control of *M. tuberculosis* infection of gut-lung immune interaction axis but also reveals a previously unidentified strategy for the development of a protective immune paradigm against deadly TB and other lung diseases.

## Materials and methods

### Methods

**Study subjects**: Subjects with newly diagnosed active TB infection but did not receive any anti-TB treatment were recruited by the Guangdong Provincial Center for TB Control in this study. All subjects were ≥18 years of age and were sero-negative for HIV-1/2, other infectious diseases and major diseases, such as cancer and diabetes. The sex ratios (female/male) of each group are healthy controls (HC) (28/15) and TB patients (TB)(29/26). The ages of each group (median [interquartile range]) were HC, 27 (24–35); TB, 34 (26–40). The healthy controls (HCs) came to the same hospital clinics for annual routine health examinations while providing fecal samples, with final reports indicating healthy statuses. Diagnosis of pulmonary TB was based on symptoms, roentgen graphic findings (chest X-ray and/or HRCT) and sputum bacillus examination. The numbers of bacilli in sputum smears were counted according to World Health Organization (WHO) guidelines. Of 55 TB patients, 48 had sputum positive culture for *M. tuberculosis* and were divided into four groups as per grading of the sputum AFB smear: group I (sputum1+, n = 9): 1 to 9 AFB per 100 fields; group II (sputum 2+, n = 8): 10 to 99 AFB per 100 fields; group III (sputum 3+, n = 8): 1 to 10 AFB per field in 50 fields; and group IV (sputum 4+, n = 5): more than 10 AFB per field in 20 fields. Sputum samples were classified according to the highest number of AFB per specimen. Fecal samples and blood samples from TB subjects were collected in sterile containers within 7 days prior to initiating standard anti-TB treatment. Informed consent from all participants included in the study was obtained according to protocols approved by the Internal Review and the Ethics Boards of Zhongshan School of Medicine of Sun Yat-sen University (SYSU).

**Mice**: Specific-pathogen-free (SPF) C57BL/6(3 ~ 4 weeks) were obtained from SYSU Experimental Animal Center. LncRNA-CGB KO mice were generated using a CRISPR genome-editing system in the C57BL/6 background. Single guide RNAs (sgRNAs) flanking region flanking exon 1 and exon 2 to exon 7 of lncRNA – CGB were designed using a CRISPR design tool (genome-engineering.org). A Cas9 expression plasmid (Addgene) was linearized with PmeI and used as a template for in vitro transcription. Purified Cas9 mRNA and sgRNAs were mixed and injected into the cytoplasm of fertilized eggs of C57BL/6 mice in the M2 medium (Sigma-Aldrich). Successful KO mice were validated by PCR with the following primers: “Pair1-F: GGCCTCTGATTTAGCCAGCACTG”; “Pair1-R: GTGTACCCCAGCATAATGGCGAT;” “Pair2-F: CAATGGCTTGACCCAGACTTAGGAG;” and“Pair2-R: GACATTCCCTGGCATTCATATCAGG”. The detected mutated allele was selected to mate with the wild-type C57BL/6 strain to obtain F1-generation mice. Heterozygous F1 offspring were interbred to establish lncRNA-CGB-/ – Strain. All procedures were carried out under approval by the SYSU Institutional Animal Care and Use Committee (Approval number SYSU-IACUC-2019-B527).

***M. tuberculosis* infection**: Mice were infected with *M. tuberculosis* (strain H37Rv) *via* aerosol challenge. To determine the *M. tuberculosis* burden at indicated times, the lung of each mouse was homogenized in PBS, and ten-fold serial dilutions were made in PBS and plated on 7H10 plates. Colonies were counted after 21d of incubation at 37°C, and CFUs per lung were determined. Approximately 100 ~ 150 CFUs of *M. tuberculosis* were deposited in the lungs of each mouse upon initial infection.

**Histopathological, bacterial and immune analyses of *M. tuberculosis-*infected mice**: At the end of the *M. tuberculosis* infection period, animals were sacrificed. The right lung and cecum were excised and carefully homogenized. For H&E and acid-fast Kinyoun’s analysis, lung or gut tissues of mice were fixed in 10% formalin and processed for paraffin embedding. Paraffin sections of 5 µm were counterstained with H&E or Kinyoun’s. Images of acid-fast staining were obtained using a microscope (Olympus BX63). H&E staining was taken by Digital Slide Scanning System AxioScan.Z1. All sections were interpreted by the same person and scored semiquantitatively, blinded to the variables of the experiment. The histopathological parameters inflammatory lesions and granuloma formation were scored as our previous study.^14^

**Antibiotics treatment**: A mix of ampicillin (1 mg/ml), neomycin (1 mg/ml), metronidazole (1 mg/ml) and vancomycin (0.5 mg/ml) were added in sterile drinking water of mice. Solutions and bottles were changed 2 ~ 3 times a week. The antibiotic activity was confirmed by macroscopic changes observed at the level of the cecum (dilatation) and by cultivating the fecal pellets resuspended in BHI+15% glycerol at 0.1 g/ml on blood agar plates for 48 h at 37°C within aerobic or anaerobic conditions.

**Mouse lncRNA and mRNA sequencing, data analysis and statistics**: Total RNA of lungs in *M. tuberculosis*-infected mice with or without the treatment of broad-spectrum antibiotics were isolated with TRIzol reagent (Thermo Fisher). After RNA cleanup and quality analysis, total RNA was used for mRNA and lncRNA high throughput sequencing analysis. Briefly, a total amount of 2 μg RNA per sample was used as input material for the RNA sample preparations. Sequencing libraries were generated using NEBNext® Ultra™ RNA Library Prep Kit for Illumina® (#E7530L, NEB, USA) following the manufacturer’s recommendations and index codes were added to attribute sequences to each sample. The details of library construction showed as follows: Firstly, ribosomal RNA was removed by kits, RNA fragmentation and short RNA strands were carried out by NEB Next First Strand Synthesis Reaction Buffer under elevated temperature. Subsequently, the first cDNA strand was synthesized using random hexamer primers and RNA fragments as a template. Second strand cDNA synthesis was subsequently performed using buffer, dNTPs, DNA polymerase I and RNase H. The library fragments were purified with QiaQuick PCR kits and elution with EB buffer, then terminal repair adds poly (A) and adapter were implemented. In order to select cDNA fragments of preferentially 300bp in length, the library fragments were purified and the UNG enzyme was used to digest the second strand of cDNA. PCR was performed, aimed products were to retrieve, and the library was completed. Afterward, the clustering of the index-coded samples was performed on a cBot cluster generation system using TruSeq PE Cluster Kit v4-cBot-HS (Illumina) according to the manufacturer’s instructions. After cluster generation, the libraries were sequenced on an Illumina platform and 150 bp paired-end reads were generated.

**Gene Set Enrichment Analysis (GSEA**): GSEA was performed using the GSEA software (http://www.broadinstitute.org/gsea) with permutation type = gene set, number of permutations = 1000 enrichment; statistic = weighted, and metric for ranking genes = Singal2Noise. GSEA for analyzing the pathway for antibiotics treatment was performed using the gene set of mouse pathway target gene sets. The heat map was generated and visualized by using the Morpheus software (https://software.broadinstitute.org/morpheus/).

**Northern blot**: Total RNA was extracted from indicated samples by Trizol (Roche) according to the manufacturer’s manual. A total of 10 μg of RNA was electrophoresed on a 10% formaldehyde gel and transferred to a Biodyne Nylon membrane (Pall). The membrane was further hybridized with a digoxin-labeled lncRNA-CGB probe (5ʹDIG – AGATGATGGTAGGATGTGCTT-3ʹDIG) (Exiqon) at 68°C overnight in ULTR AhybTM-Oligo buffer. U6 probe and Scramble probe were used as a positive and negative control, respectively. The membrane washes were performed as described in the Northern Max Kit protocol (Roche) and the signals were visualized using an Odyssey infrared scanner (Li-Cor).

**DNA extraction, 16S rDNA gene amplification, and pyrosequencing (human fecal samples**): Fresh fecal stool samples were collected from patients with active TB disease and healthy controls, respectively. DNA was isolated with the DNA STOOL Kit (QIAGEN) followed the manufacturer’s instructions. The purity, concentration of DNA was detected by using the NanoPhotometer spectrophotometer and Qubit2.0 Fluorometer. DNA was then amplified by the corresponding primer: the 338 F-533 R for the V3 Regions, the 341 F-805 R for V3+ V4 regions and the 967 F-1046 R for the V6 regions. The index sequences were added and enriched after the extraction finished. The Qubit2.0, Agilent 2100 and Bio-rad CFX 96 were used to quantify the concentration and purity of the library to ensure the quality. After all had finished, the library was sequenced on an Illumina HiSeq2500 by using the 250 paired-end protocol.

**Alpha diversity analysis**: Alpha diversity was implemented to display the diversity of bacteria among the different samples, which mainly included 4 indices: Observed-species, Chao1, Shannon and Simpson. All these indices in our samples were calculated with QIIME (qiime-1.8.0) and displayed by R software.

**OTU abundances analysis**: To investigate differences in OTUs, genera, species abundances between both groups, raw counts were normalized then log-transformed using the normalization method below, as performed by a previous study:^70^

Normalized count = log10 ((raw count/ number of sequences in that sample) × average number of sequences per sample+1).

Wilcoxon tests were used to compare mean differences between two groups for genera and species log abundances. Considering t = total number of taxa tested, p = raw *p*-value and R = sorted rank of the taxon, *P*-values were corrected for multiple testing ^70^ using:

Adjusted p-value = t × p R Fold changes for each genus/OTU were calculated using:

Log2 FC = log2 (TB average + 1) − log2 (HC average + 1)

**Monoclonal antibodies (mAbs) for intracellular cytokine staining (ICS**): The following antibodies were used for flow cytometric assay: mouse anti-CD4 APC-eFluor 780(GK1.5, eBio), mouse anti – CD8-FITC (53–6.7, eBio), mouse anti-IFN-γ APC (XMG1.2, eBio).

**Flow cytometric analysis**: Experiments were conducted as described in our previous publication.^28^ Briefly, cells were first stained with cell-surface markers and then fixed and permeabilized for 30 min before staining the intracellular molecules for another 45 min. Finally, cells were fixed with 2% formalin/PBS and processed for polychromatic flow cytometry. Isotype matched IgG staining was served as a negative control. Data were obtained from Beckman Coulter Gallios and analyzed by Kaluza 1.5 software.

**Bacterial culture**: *Bacteroides fragilis* (purchased from CCTCC, Wuhan, China) were grown on blood agar plates or selective agar plates for 48 hours at 37°C in anaerobic conditions. Bacteria were harvested from the agar plates, suspended in sterile saline with 10% glycerol, centrifuged and washed once with saline, then resuspended in sterile saline at an optical density (600 nm) of 1, which corresponded approximately to 1 × 10^9^ colony-forming units (CFU)/ml. The bacterial culture was aliquoted and stored at −80°C for future use.

**Bacteria oral transfer**: SPF mice receiving antibiotics for 1 week were orally gavaged daily with 1 × 10^9^ CFU of *B. fragilis* or saline until death. Colonized mice with dedicated bacteria were subjected to *M. tuberculosis* infection 1-week post bacteria oral transfer.

**Fecal microbial transplantation (FMT**): Fecal materials from healthy controls used for 16s sequencing were pooled by sterile saline supplemented with 15% glycerol and homogenized. The homogenate was centrifuged at 1000 g for 10 min at 4°C. The supernatant was aliquoted and stored at −80°C for future use. Antibiotics-treated mice were used for the colonization with fecal microbiota. FMT was performed by thawing the fecal materials and 0.2 ml of the pooled suspension containing fecal microbiota obtained from healthy controls was transferred by oral gavage into each recipient.

**Quantification of bacteria by qRT-PCR**: Genomic DNA was isolated from fecal samples using the QIAamp DNA Stool Mini Kit (Qiagen) following the manufacturer’s instructions. Targeted qRT-PCR systems were applied using either Taqman technology targeting the All Bacteria domain or SybrGreen for *Bacteroides fragilis* species. The primers used were summarized in Table 2.

**Table 2.** The list of qPCR primer couples

Target group		Primer sequence (5’-3’)
*Total bacteria*	F	ACTCCTACGGGAGGCAGCAGT
	R	ATTACCGCGGCTGCTGGC
*Prevotella*	F	CCTWCGATGGATAGGGGTT
	R	CACGCTACTTGGCTGGTTCAG
*Veillonella*	F	GRAGAGCGATGGAAGCTT
	R	CCGTGGCTTTCTATTCC
*Haemophilus*	F	AGCGGCTTGTAGTTCCTCTAACA
	R	CAACAGAGTATCCGCCAAAAGTT
*Blautia*	F	GTGAAGGAAGAAGTATCTCGG
	R	TTGGTAAGGTTCTTCGCGTT
*Neisseria*	F	CTGTTGGGCARCWTGAYTGC
	R	GATCGGTTTTRTGAGATTGG
*Fusobacterium*	F	AAGCGCGTCTAGGTGGTTATGT
	R	TGTAGTTCCGCTTACCTCTCCA
*Desulfovibrio*	F	GGTACCTTCAAAGGAAGCAC
	R	GGGATTTCACCCCTGACTTA
*Atopobium*	F	ACCGCTTTCAGCAGGGA
	R	ACGCCAATGAATCCGGAT
*Bifidobacterium*	F	CGGGTGAGTAATGCGTGACC
	R	TGATAGGACGCGACCCCA
*Bacteroides*	F	GAGAGGAAGGTCCCCCAC
	R	CGCTACTTGGCTGGTTCAG
*Lactobacillus*	F	AGCAGTAGGGAATCTTCCA
	R	CACCGCTACACATGGAG
*Enterococcus*	F	CCCTTATTGTTAGTTGCCATCATT
	R	ACTCGTTGTACTTCCCATTGT
*Campylobacter*	F	GGATGACACTTTTCGGAG
	R	AATTCCATCTGCCTCTCC
*Bacteroides fragilis*	F	TCRGGAAGAAAGCTTGCT
	R	CATCCTTTACCGGAATCCT

**Adoptive transfer**. CD3 + T cells and CD14+ monocytes were purified from PBMC of active TB patients using positive selection using a MACS Pro Separator (Miltenyi Biotech). Cell purity was consistently ≥95%. Three days before transfer, four-week-old SCID mice were infected with *M. tuberculosis* (H37RV). 5 × 10^5^ CD3 + T cells were transduced with LV-lncRNA-CGB or LV-Ctrl for five days. Culture supernatants for CD3 + T cells, and CD3 + T cells transduced with indicated LV vector and 4 × 10^4^ autologous monocytes were transferred into each of recipient mice *via i.p*. injections.

**Western blotting**: Cells isolated from indicated mice were seeded. Total protein was extracted from cells using RIPA lysis buffer. Extracted proteins were mixed with loading buffer, subject to 10% SDS-PAGE and transferred to PVDF membranes, which were subsequently blocked in a 5% solution of nonfat milk for 1 hour. The membranes were subsequently incubated with the following antibodies overnight at 4°C: rabbit anti-H3K9Me1(ab9045,Abcam), mouse anti-H3K27Me3(ab6002,Abcam), rabbit anti – H3K4Me3(ab8580,Abcam), rabbit anti-H3(ab1791,Abcam),mouse anti-β actin (ab6276,Abcam), rabbit anti-H3K27Me2(ab24684,Abcam), mouse anti – H3k9Me2(ab1220, Abcam), rabbit anti-H3K9Me3(ab8898,Abcam).The membranes were further incubated with goat anti-rabbit or goat anti-mouse secondary antibody (Thermo Fisher) for 1 hour at room temperature. Signals were detected with Western Lightning Plus-ECL Enhanced Chemiluminescence reagent. Images were captured by the BIO-RAD ChemiDoc Touch machine.

**Isolation of peripheral blood mononuclear cells (PBMCs**): PBMCs were obtained by density centrifugation of diluted blood (1-part blood to 1-part PBS) over Ficoll-Paque. Cells were washed twice in PBS and suspended in the medium (RPMI 1640) supplemented with 10%FBS and were cultured for future use in a 96 well plate.

**RNA interference**: Control siRNA and siRNA target lncRNA-CGB were synthesized by Exiqon. siRNA transfections were performed using Lipofectamine RNAi MAX (Thermo Fisher) following the manufacturer’s instructions. The final concentration of the siRNA molecules is 20 nM and cells were stimulated with or without *M. tuberculosis* lysates for 48 hours according to the purposes of the experiments. Transfected cells were analyzed by qRT-PCR to analyze the expression of lncRNA-CGB post-transfection to determine transfection efficiency.

**FISH and immunofluorescence of lncRNA-CGB, EZH2 in PBMCs**: PBMCs were fixed 4% paraformaldehyde for 20 min at RT, and further permeabilized by 70% ethanol for another 2 hours. PBMCs were then incubated with rabbit anti-EZH2 with digoxin labeled lncRNA-CGB in hybridization buffer overnight at 37°C, respectively. Nonspecific binding of probes was removed by a subsequent wash with washing buffer. Cells were further stained with goat anti-rabbit-FITC and anti-digoxin-Rhodamine at 37°C for another 30 min. ProLong Gold Antifade with DAPI was used for nuclear counterstain and cells were air-dried for further microscopic analysis by using a confocal microscope (Zeiss 780).

**Chromatin Immunoprecipitation and ChIP-qPCR**: 1 × 10^6^ cells were prepared for each round of ChIP. ChIP was performed using EZ MAGNA ChIP assay kit (millipore) according to the manufacturer’s manual. Briefly, after fixation of cells with formaldehyde (1%), Chromatin DNA was immunoprecipitated with the following antibodies: rabbit anti-H3K9Me1 (ab9045, Abcam), mouse anti-H3K27Me3 (ab6002, Abcam), rabbit anti-H3K4Me3 (ab8580, Abcam), rabbit anti-H3 (ab1791, Abcam), rabbit anti-H3K27Me2 (ab24684, Abcam), mouse anti-H3k9Me2 (ab1220, Abcam), rabbit anti-H3K9Me3 (ab8898, Abcam). PCRs were performed in 20-μl reaction mixtures containing 2 μl of DNA, 10 μl of SYBR green master mix and 1 μl of each primer. The following gene-specific ChIP-qPCR primers were used:

**Table:** 

Gene	Position	Oligo sequence 5’-3’(Forward)	Oligo sequence 5’-3’(Reverse)
*ifng*	A	ATGGAGGAACTAGATGATT	CTTCAATGGAGGTGATTAG
*ifng*	B	GGTCAAGATAACTGGGTA	ATTCCTAGTGGCTCAATT

The PCR amplimers were confirmed to be a single band of the correct size by agarose gel electrophoresis. ChIP-qPCR data were normalized by the % input method. Results are expressed as means ± SEM of the indicated number of independent determinations.

**RNA Immunoprecipitation**: RIP was performed using the Magna RIP-Binding Protein Immunoprecipitation Kit (Millipore). Briefly, anti-EZH2 (AB3748; Abcam), anti-PRDM16 (ab106410, Abcam), anti-G9a (ab40542; Abcam) or control IgG were first incubated with Protein G dynabeads for 30 min, isolated PBMCs of TB patients or lymphocytes of lungs of *M. tuberculosis*-infected mice were lysed followed by incubation with antibody-dynabeads complexes overnight, immunoprecipitated RNA was then purified according to the manufacturer’s protocol. LncRNA-CGB and control RNAs were then analyzed by PCR and gel electrophoresis.

**Reverse transcription qRT-PCR (qPCR**): Total RNA was prepared from tissues or PBMCs using Trizol reagent (Takara). The concentration of total RNA was determined by a Nanodrop spectrometer. First-strand cDNA was synthesized using the PrimeScriptone step Strand cDNA Synthesis Kit (Takara) following the manufacturer’s instructions; qPCR was performed in technical triplicate using the SYBR Green to determine the expression of IFN-gamma and lncRNA-CGB. GADPH was used as an endogenous control to normalize gene expression. Relative mRNA expression levels were presented as means ± SEM. Statistical differences were analyzed by the Student’s *t*-test.

**IFN-γ-ELISPOT analyses**: ELISPOT plates (Millipore, MAIP S4510) were coated with purified IFN-γ (BD) antibody overnight at 4°C followed by blocking with 10% FBS in RMPI1640 for 2 hours at room temperature. Cells derived from indicated mice were plated at 1 × 10^6^ cells per well and stimulated with ESAT-6 (6 kDa Early Secretory Antigenic Target) peptides, CFP-10 (10-kDa Culture Filtrate Protein) peptides and *M. tuberculosis* lysates. Spots were developed using the BD mouse IFN-γ kit (Cat. No. 551083), and the number of spots was measured using an Immunospot Series 3 Analyzer and analyzed using ImmunoSpot software.

**Cytometric bead assay (CBA) analysis of Th1/Th2/Th17 cytokine expression**: Cells derived from mice or isolated PBMC of TB patients with indicated treatments were seeded at a density of 1 × 106 cells per well and stimulated with ESAT-6 peptides, CFP-10 peptides and *M. tuberculosis* lysates at 37°C, respectively. Culture supernatants of cells were analyzed for the production of the following cytokines: IL-2, IL-4, IL-6, IFN-γ, TNF-α, IL-17A and IL-10 using the cytometric bead assay (CBA) mouse Th1/Th2/Th1 cytokine kit (BD) according to the manufacturer’s instructions. Data were acquired on the Beckman Coulter Gallios (Beckman). The concentrations of each cytokine were revealed by the fluorescence intensity. Cytokine concentrations were calculated relative to the standard dilution curve.

**Bioinformatics analyses of evolutionary conservation and coding potential of lncRNA-CGB and plasmid constructions**: Evolutionary conservation of lncRNA-CGB was analyzed using the University of California Santa Cruz (UCSC) Genome Browser (genome.ucsc.edu). The coding potential of lncRNA-CGB was first analyzed using the tools provided by the Peking University Center for Bioinformatics (cpc.cbi.ku.edu.cn/programs/run_cpc.jsp) and further constructed into the ORF of eukaryotic expression vector pReceiver-M98 with EGFP tag. Vector pReceiver-M98R with only EGFP tag or and vector pReceiver-M98Rwith EZH2 and EGFP tag were served as controls. All constructs were confirmed by DNA sequencing.

**In silicon prediction for RNA structure and protein-RNA interaction**: RNA secondary structure was predicted by RNA fold Webserver (http://rna.tbi.univie.ac.at/cgi-bin/RNAWebSuite/RNAfold.cgi) based on minimum free energy (MFE) and partition function. The binding propensity between EZH2 and RNA fragments was predicted by catRAPID (http://service.tartaglialab.com/page/catrapid_group).

**Statistical analysis**: Statistical analysis was performed using GraphPad Prism. Unless otherwise indicated, the non-parametric Mann-Whitney U-test was often used for two groups comparisons to avoid the assumption of a normal distribution. For samples more than 20, statistical analyses were performed with the unpaired two-tailed *Student t-test* as indicated in the figures. *p-*value <0.05 were considered significant. *p < .05, **p < .01, ***p < .001 and **** *p* < .0001. NS, no statistical significance. All experiments were replicated in the laboratory at least two times. In figure legends, N represents the number of samples.

## Supplementary Material

Supplemental Material

## Data Availability

The authors confirm that the data supporting the findings of this study are available within the article and its supplementary materials. The datasets generated for this study can be found in the GSE125870.https://www.ncbi.nlm.nih.gov/geo/query/acc.cgi?acc=GSE125870
