# Supplementary material for: The gut microbiota mediates protective immunity against tuberculosis via modulation of lncRNA
Source: Gut Microbes. 2022 Mar 28;14(1):2029997. doi: 10.1080/19490976.2022.2029997 (PMC8966992; doi:10.1080/19490976.2022.2029997)
Supplement: Supplemental Material [file KGMI_A_2029997_SM9061.zip › Supplementary information/Supplementary_Gut_microbes_KGMI_20210490R1.docx]

**Supplementary Information Figures 1-14**

**Supplementary Figure 1. Antibiotics induced dysbiosis in the gut in mice.**

The mean relative abundance at Genus of gut microbiota with/without antibiotics-treatment in mice. Broad-spectrum antibiotics effectively reduced the compositions of gut microbiota. N = 6 per group.

# Supplementary Figure 2. LncRNA-CGB has no coding capability.

(a) Diagram of lncRNA-CGB.

(b) The conservation track of lncRNA-CGB analyzed by UCSC Genome Browser ([http://genome.ucsc.edu/).](http://genome.ucsc.edu/)) The human and mouse lncRNA-CGB share 51.5% homologous sequences.

(c) The analysis of coding potential of lncRNA-CGB using tools provided by <http://cpc.cbi.pku.edu.cn/programs/run_cpc.jsp> showed that lncRNA-ENST00000434839 was lack of coding potential.

(d) Gel electrophoresis of lncRNA-ENST00000434839 extracted from nucleus and cytoplasm of CD3+ T cells purified from PBMCs of patients with active TB. As controls, more β-actin expressed in the cytoplasm and more U6 expressed in the nucleus, respectively.

(e) Fluorescent imaging showed that EZH2-eGFP plasmid, eGFP plasmid, lncRNA- ENST00000434839-eGFP plasmid, and lncRNA-ENST00000434839 plasmid, as schematically shown in the left panel, did not express eGFP after transfecting to HEK293T cells.

(f) Immunoblotting using the antibody specific to eGFP showed that lncRNA- ENST00000434839-eGFP plasmid and lncRNA-ENST00000434839 plasmid did not express GFP after transfecting to HEK293T cells.

# Supplementary Figure 3. Confirmation of specificity of northern blot.

Northern blotting analysis of lncRNA-CGB expression using control probe in splenocytes isolated from *M. tuberculosis*-infected wild-type mice with drinking antibiotics and water only.

**Supplementary Figure 4. *M. tuberculosis* infection reduced the expression levels of lncRNA-CGB in mice.**

qPCR analysis of lncRNA-CGB expression of PBMCs derived from *M. tuberculosis-*infected and uninfected mice. N=6 per group. **p<*0.05.

# Supplementary Figure 5. Construction and validation of lncRNA-CGB knock-out mice.

LncRNA-CGB knock-out (KO) mice were generated by CRISPR/Cas9 technology in the C57BL/6 background.

(a) Genomic region of mouse lncRNA-CGB (Gene ID: 213742; Ensembl: ENSMUSG00000086503) locus was diagrammed (gene was oriented from left to right; total size was 22.86 kb). Solid bars represent ORF; open bars present UTRs. Eight exons have been identified). Single guide RNAs (sgRNAs) flanking region flanking exon 1 and exon 2 to exon 7 of lncRNA-CGB were designed using a CRISPR design tool (genome- engineering.org). A Cas9 expression plasmid (Addgene) was linearized with PmeI and used as a template for *in vitro* transcription. Purified Cas9 mRNA and sgRNAs were mixed and injected into the cytoplasm of fertilized eggs of C57BL/6 mice in M2 medium (Sigma-Aldrich).

(b) Successful knockout mice were validated by PCR with agarose gel. The detected mutated allele was selected to mate with the wild-type C57BL/6 strain to obtain F1 offspring lncRNA-CGB KO mice.

**Supplementary Figure 6. Adoptive transfer of lncRNA-CGB-depressed CD3+ T cells to *M. tuberculosis*-infected SCID mice increased *M. tuberculosis* infection and TB pathology compared to lncRNA-CGB-expressed controls.**

CD3+T cells and CD14+ monocytes were first purified from PBMC of active TB patients for adoptive transfer experiments in mice, and purified CD3+ T cells were transduced with lentiviral vector encoding shRNA targeting lncRNA-CGB (sh-lncRNA-CGB) or lentiviral vector only (shRNA-Ctrl). CD3+T cells with indicated transduction and autologous monocytes were adoptively transferred to *M. tuberculosis*-infected mice.

(a) *M. tuberculosis* burdens (CFU, H37Rv) in the lungs of mice with adoptive transfer of human CD3+T cells with transduction of indicated shRNA.

(b) H&E-stained lung sections derived from two representative mice in each group of mice. Red arrows mark the infiltration of inflammatory cells. The magnification is shown in the lower right in each image.

N=6 mice for each group of mice for each experiment with at least two biological repeats. Error bars indicate average values±SEM, **p*<0.05.

**Supplementary Figure 7. The alteration of gut microbiota diversity and abundance in normal mice during *M. tuberculosis* infection, compared with uninfected mice.**

The mean relative abundance at Genus of gut microbiota in *M. tuberculosis*-infected and uninfected mice. N = 6 per group.

**Supplementary Figure 8. No significant histological changes in the gut mucosal were observed in *M. tuberculosis*-infected lncRNA-CGB knock-out/wild-type mice with/without oral treatment of *B. fragilis* and their matched controls.**

H&E-stained cecum sections derived from *M. tuberculosis*-infected lncRNA-CGB knock-out/wild type mice with/without oral treatment of *B. fragilis* and their matched controls. N = 6 per group.

**Supplementary Figure 9. The correlation between the relative abundance of *B. fragilis* and the smear grades in sputum smear-positive TB patients.**

The detection data of 30 patients with active TB infection were subjected to analyze the correlation between smear grades and *B. fragilis* abundance in fecal samples. From this result, the relative abundance of *B. fragilis* was decreased as the smear grades increasing (r=-0.9531).

# Supplementary Figure 10. LncRNA-CGB is required for *M. tuberculosis*-specific expression of IFN-γ during active TB infection in humans.

Representative flow cytometric dot plots and bar graphic analysis of IFN-γ expression in CD4+ and CD8+T cells in intestines derived from *M. tuberculosis*-infected mice with drinking broad-spectrum antibiotics or water only. Note that intestine homogenates were directly subjected to ICS/Flow cytometry analysis to analyze the IFN-γ expression without *ex vivo* antigenic or anti-CD3/anti-CD28 restimulation. Thus, this analysis method might reveal *de novo* IFN-γ response.

Error bars indicate average values±SEM, ***p*<0.01, NS: no statistical significance.

**Supplementary Figure 11. Oral administration with *B. fragilis* enhances *M. tuberculosis*-specific expression of IFN-γ.**

(a) Representative CBA/flow cytometric fluorescent clustering and bar graphic analysis of IFN-γ expression in culture supernatants of lymphocytes of lungs isolated from mice with oral administration with saline and *B. fragilis* (1×109). Lymphocytes (1×106/culture well) were cultured in the presence or absence of the whole *B. fragilis* lysates (5μg/ml) for 3 days and subjected to CBA/flow cytometric analysis of IFN-γ expression.

(b) Representative CBA/flow cytometric fluorescent clustering and bar graphic analysis of IFN-γ expression in culture supernatants of lymphocytes of lungs isolated from mice with oral administration with saline and *B. fragilis* (1×109). Lymphocytes (1×106/culture well) were cultured in the presence or absence of *M. tuberculosis* lysates (5μg/ml) for 3 days and subjected to CBA/flow cytometric analysis of IFN-γ expression.

N=3 mice for each group of mice for each experiment with at least two biological repeats. Error bars indicate average values±SEM, ***p*<0.01, ****p*<0.001, *****p*<0.0001, NS: no statistical significance.

# Supplementary Figure 12. LncRNA-CGB comprised of the motif with high interaction potency with EZH2.

(a) Predicted secondary structures of a 51-mer motif in mouse lncRNA-CGB an 89-mer motif in mouse lncRNA-*Hotair*.

(b) Predicted interaction scores of EZH2 with lncRNA-CGB and lncRNA-*Hotair*, respectively, determined by online software catRAPID. Y axis represents the interaction scores, and X axis represents the nucleotide position of the indicated lncRNA.

(c) Comparison in terms of interaction potency between EZH2/lncRNA-CGB and EZH2/ lncRNA-*Hotair*. Scores for Interaction Propensity, Discriminative Power, Normalized Interaction Score in three different regions of EZH2 and three different regions of lncRNA- CGB or lncRNA-*Hotair* were generated using catRAPID.

**Supplementary Figure** **13. Weak interaction between lncRNA-CGB and EZH2 in PBMCs derived from healthy controls.**

(a) Confocal microscopic images of RNA FISH assay of lncRNA-CGB and immunofluorescence analysis of EZH2 show that few co-localization between EZH2 with lncRNA- CGB in the nucleus of T cells isolated from healthy controls. More than 20 cells were examined and had similar results.

(b) Gel electrophoresis analysis of lncRNA-CGB retrieve in immunoprecipitation (IP) by IgG, anti-EZH2, anti-PRDM16, or anti-G9a antibody in lysates of T cells isolated from PBMC of healthy controls.

**Supplementary Figure 14. Hypothetic working model of regulating anti-TB protective immune paradigm via manipulating commensal bacteria-governed gut-lung immune interaction axis.**

Active TB in patients is associated with altered abundance, composition, diversity of gut microbiota, and aberration of gut microbiota contributes to TB pathogenesis. LncRNA-CGB, which is modulated by a more abundant commensal gut bacteria (i.e. *B. fragilis*), interacts with EZH2 for inhibiting EZH2 translocation to the IFN-γ (*ifng*) promoter and antagonizing H3K27Me3 at *ifng* promoter, and therefore allows or sustains IFN-γ expression and immunity against TB. Thus, targeting gut microbiota may serve as a novel strategy for achieving enhanced immune protection against TB.
