# Supplementary figures and images for: The gut microbiota mediates protective immunity against tuberculosis via modulation of lncRNA
Source: Gut Microbes. 2022 Mar 28;14(1):2029997. doi: 10.1080/19490976.2022.2029997 (PMC8966992; doi:10.1080/19490976.2022.2029997)

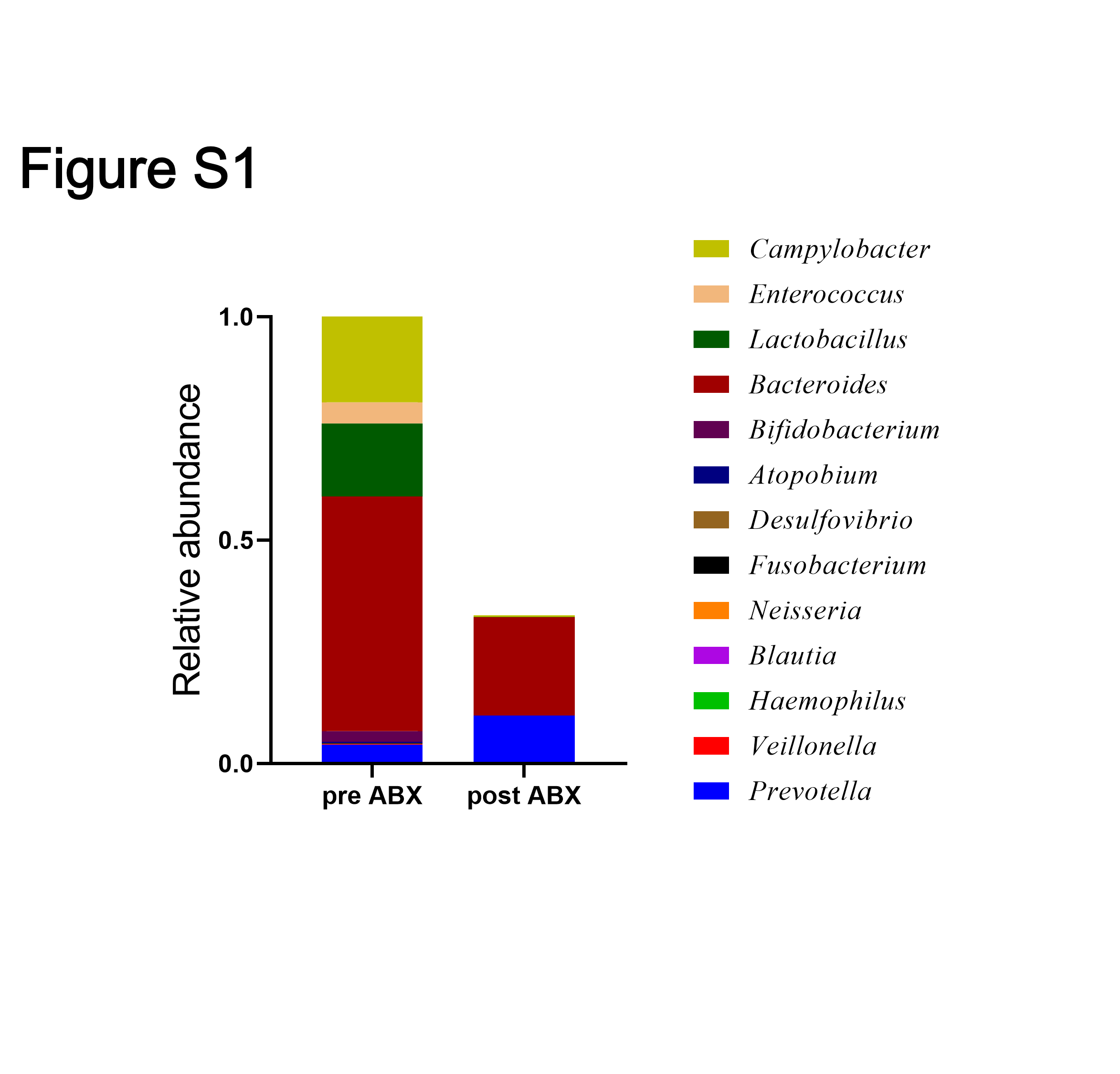

Supplement: Supplemental Material [file KGMI_A_2029997_SM9061.zip › Supplementary information/Supplementary Figure 1.TIF]

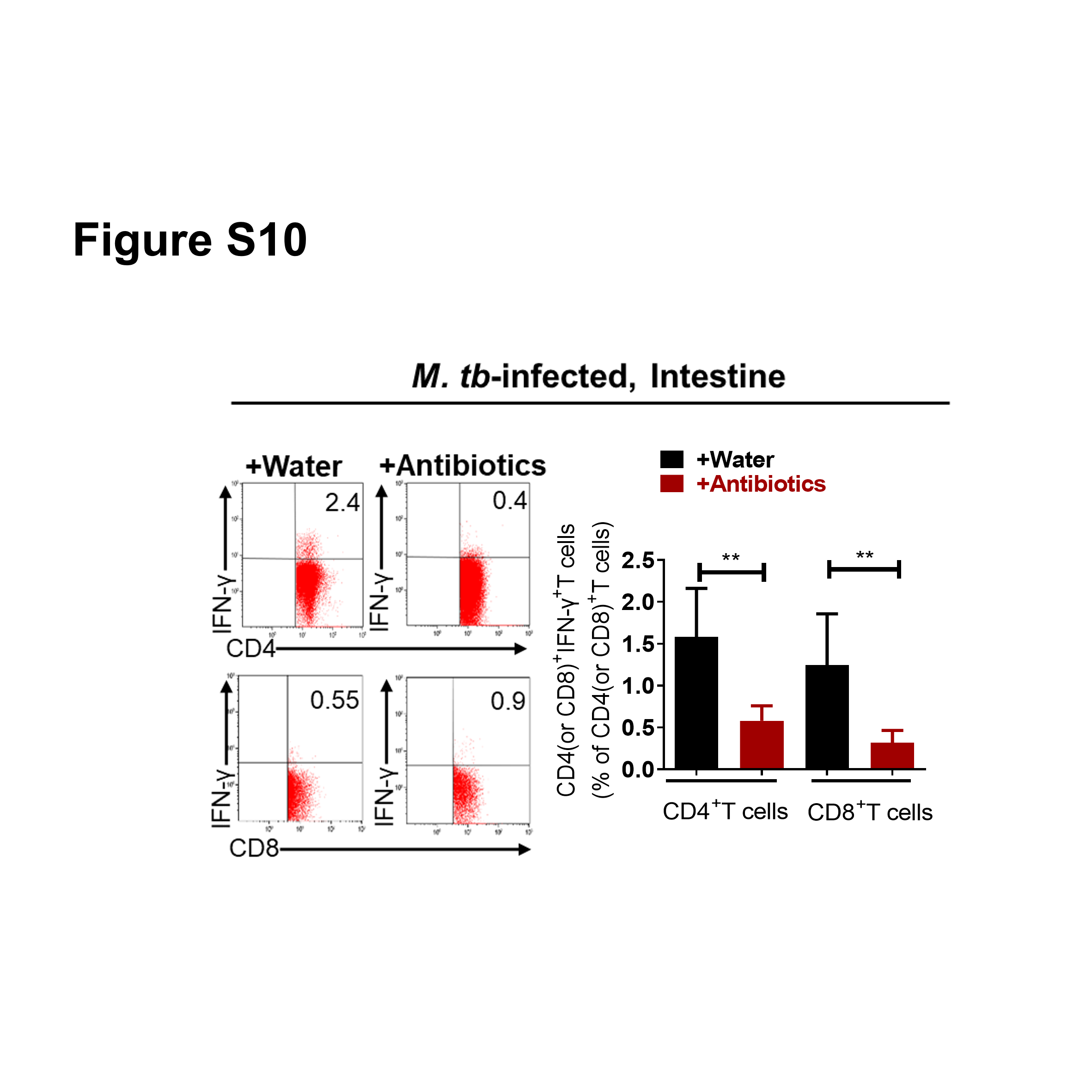

Supplement: Supplemental Material [file KGMI_A_2029997_SM9061.zip › Supplementary information/Supplementary Figure 10.tif]

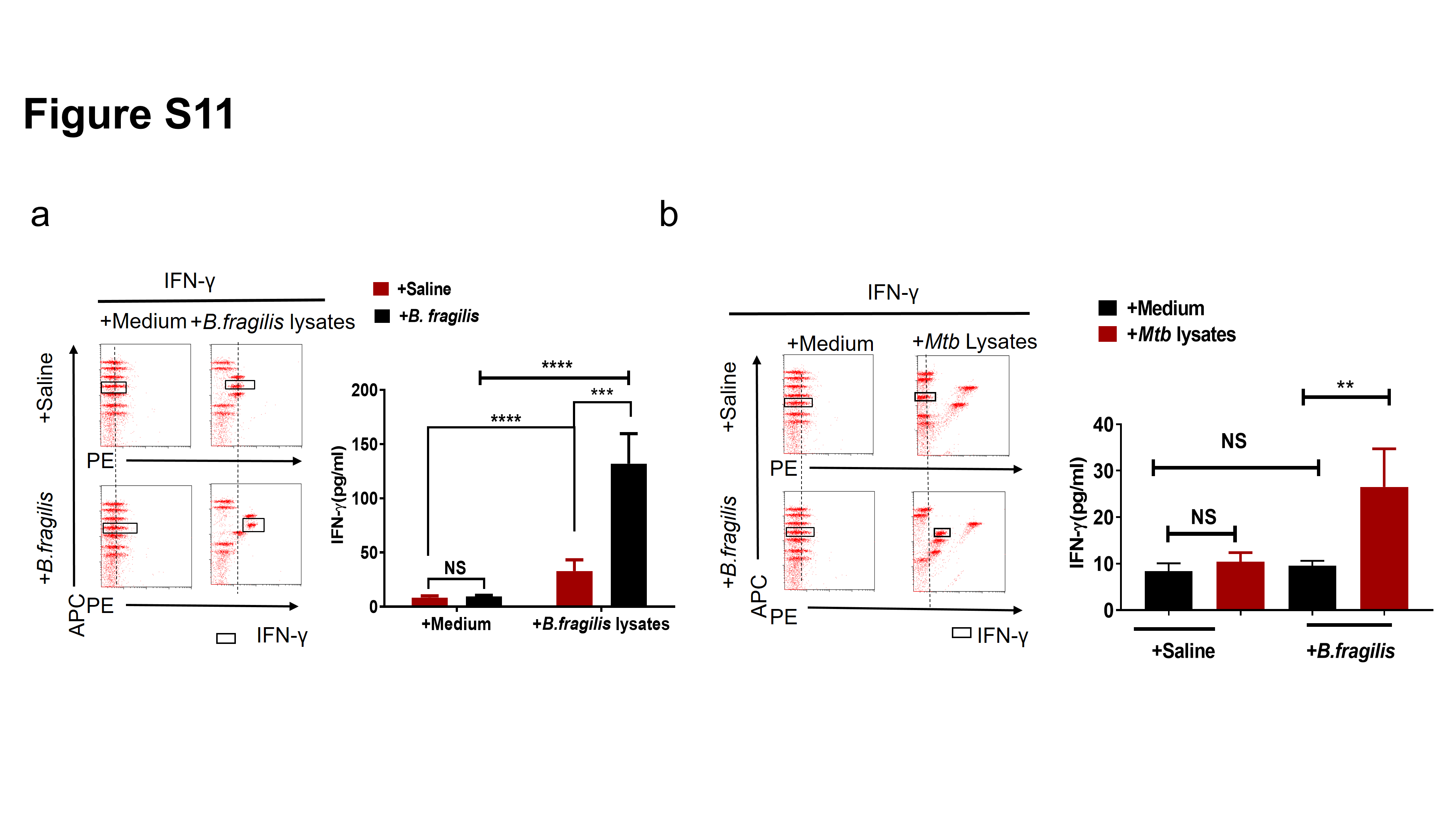

Supplement: Supplemental Material [file KGMI_A_2029997_SM9061.zip › Supplementary information/Supplementary Figure 11.tif]

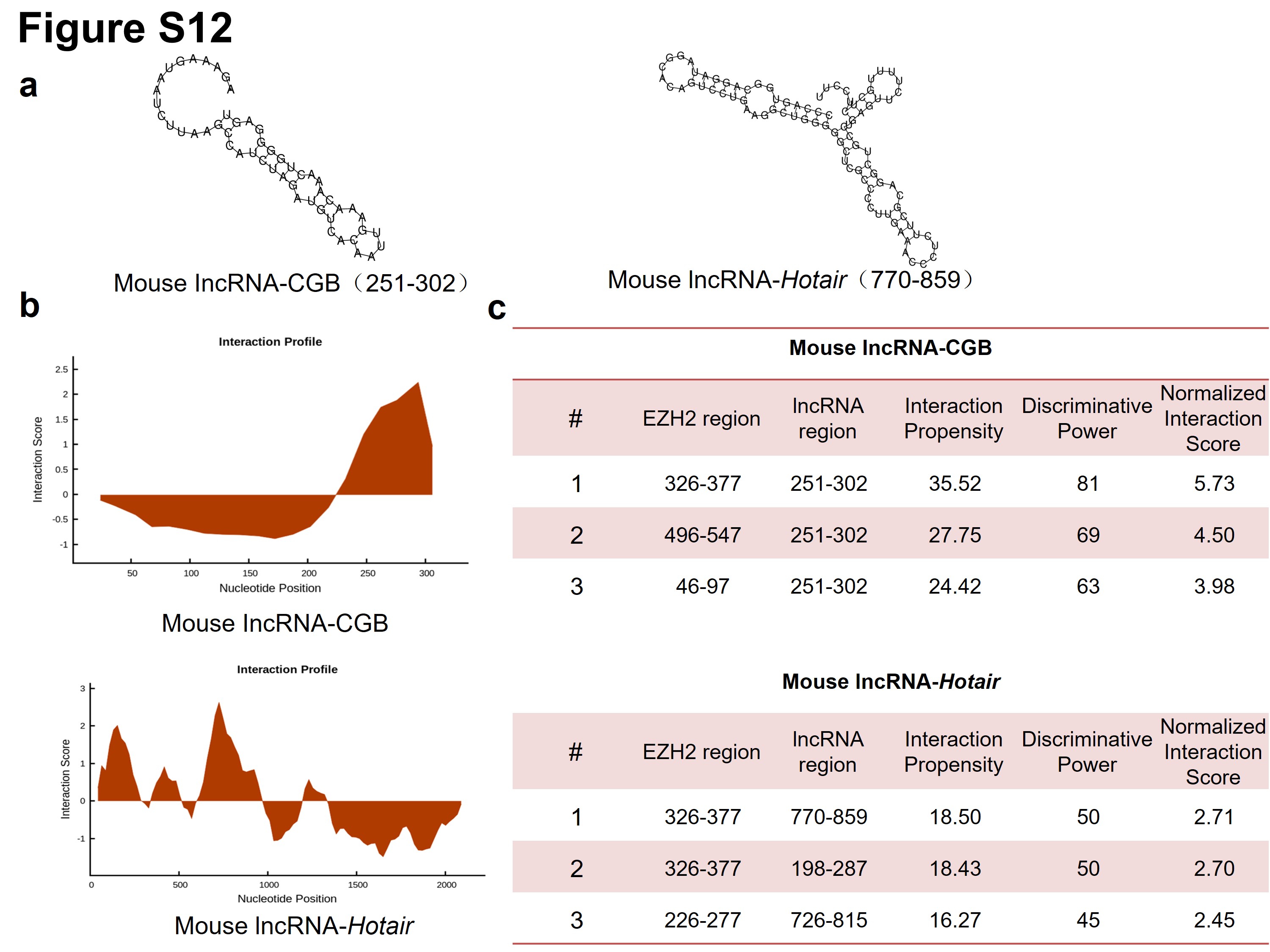

Supplement: Supplemental Material [file KGMI_A_2029997_SM9061.zip › Supplementary information/Supplementary Figure 12.tif]

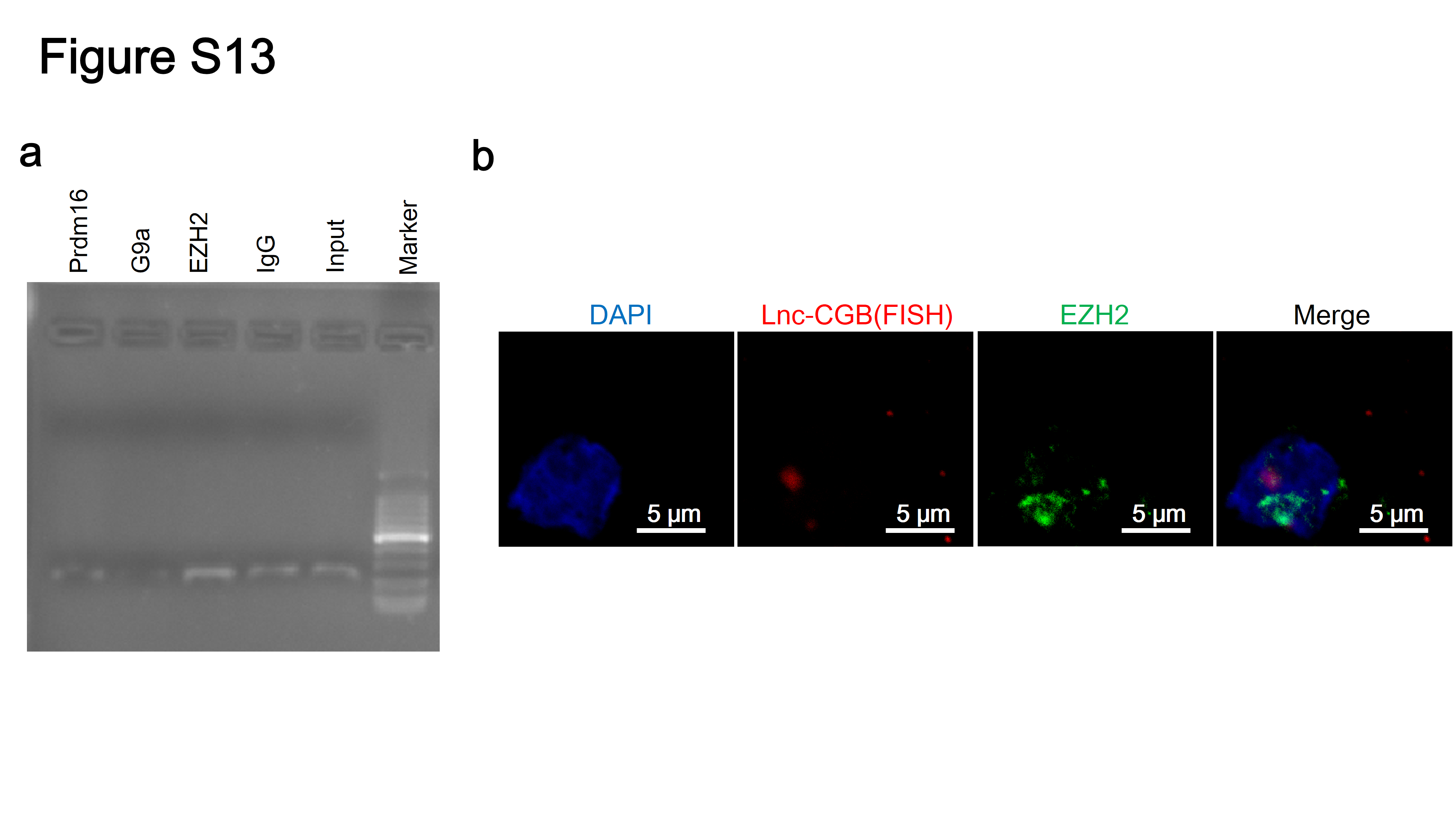

Supplement: Supplemental Material [file KGMI_A_2029997_SM9061.zip › Supplementary information/Supplementary Figure 13.tif]

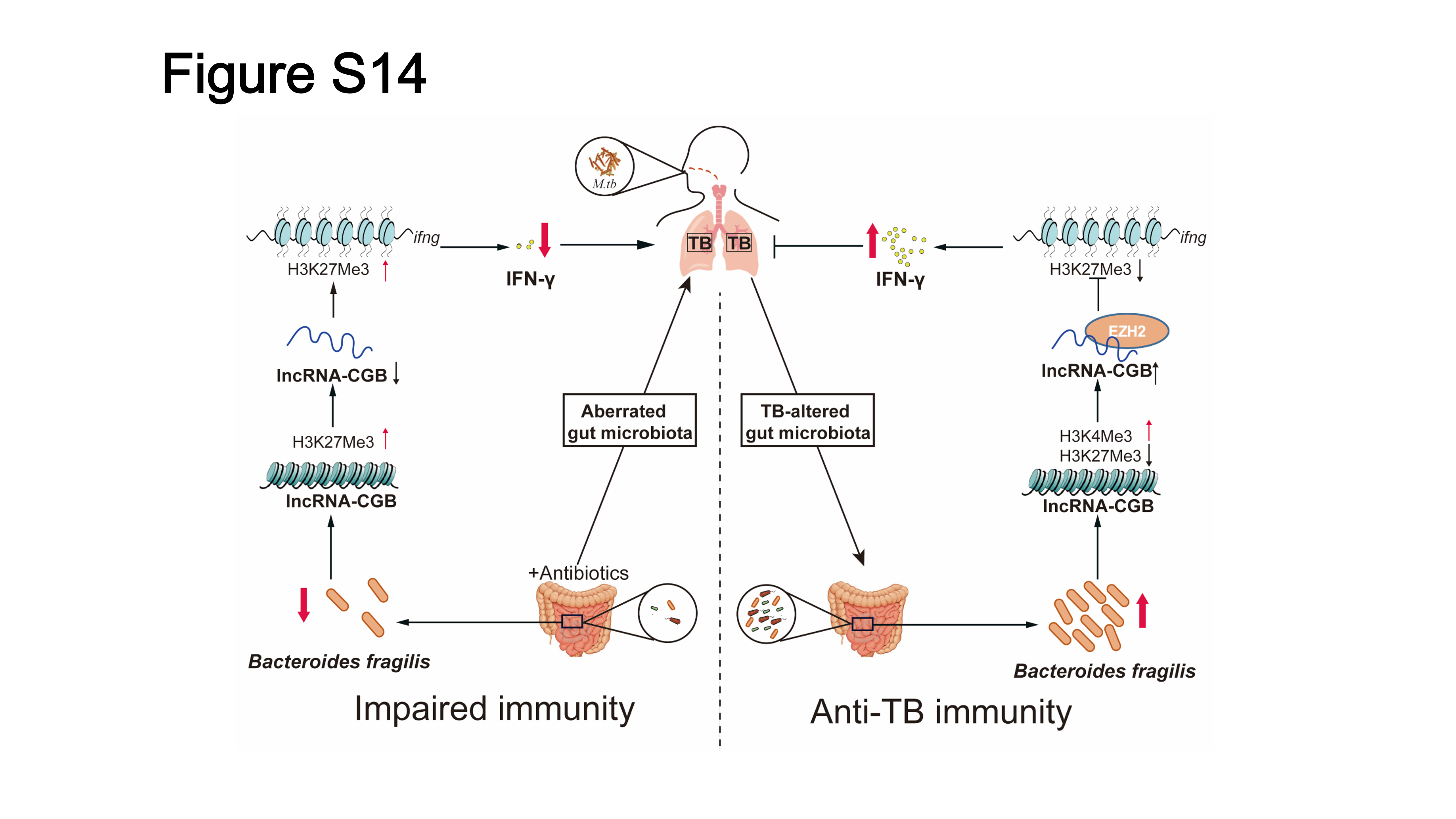

Supplement: Supplemental Material [file KGMI_A_2029997_SM9061.zip › Supplementary information/Supplementary Figure 14.tif]

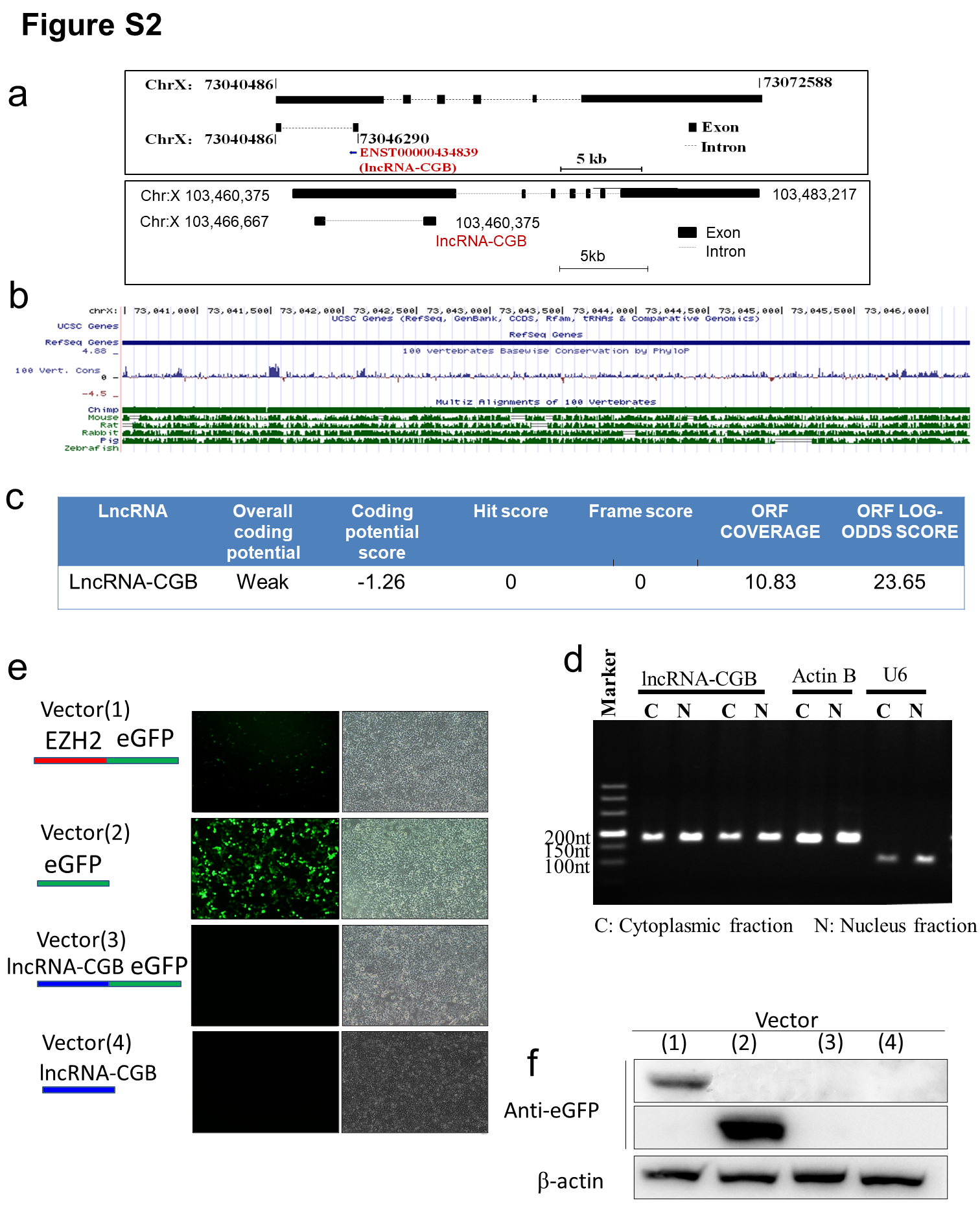

Supplement: Supplemental Material [file KGMI_A_2029997_SM9061.zip › Supplementary information/Supplementary Figure 2.tif]

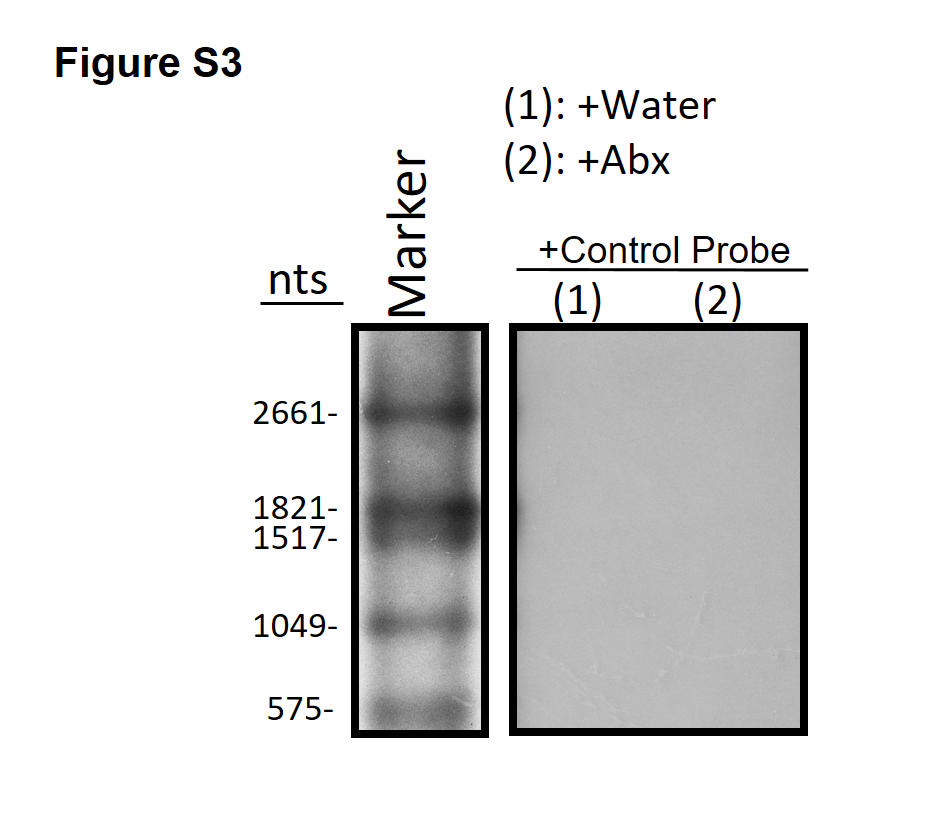

Supplement: Supplemental Material [file KGMI_A_2029997_SM9061.zip › Supplementary information/Supplementary Figure 3.tif]

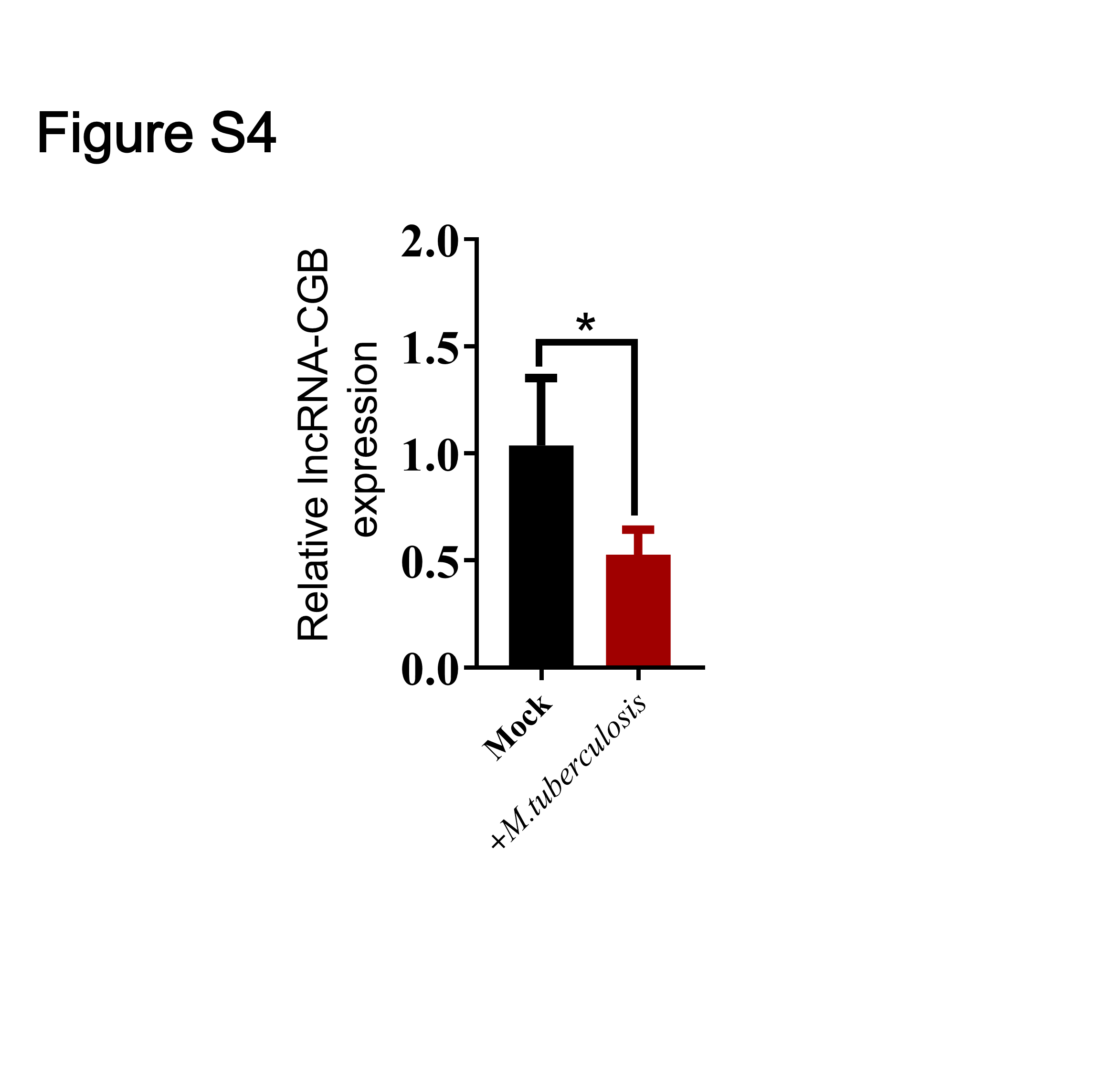

Supplement: Supplemental Material [file KGMI_A_2029997_SM9061.zip › Supplementary information/Supplementary Figure 4.TIF]

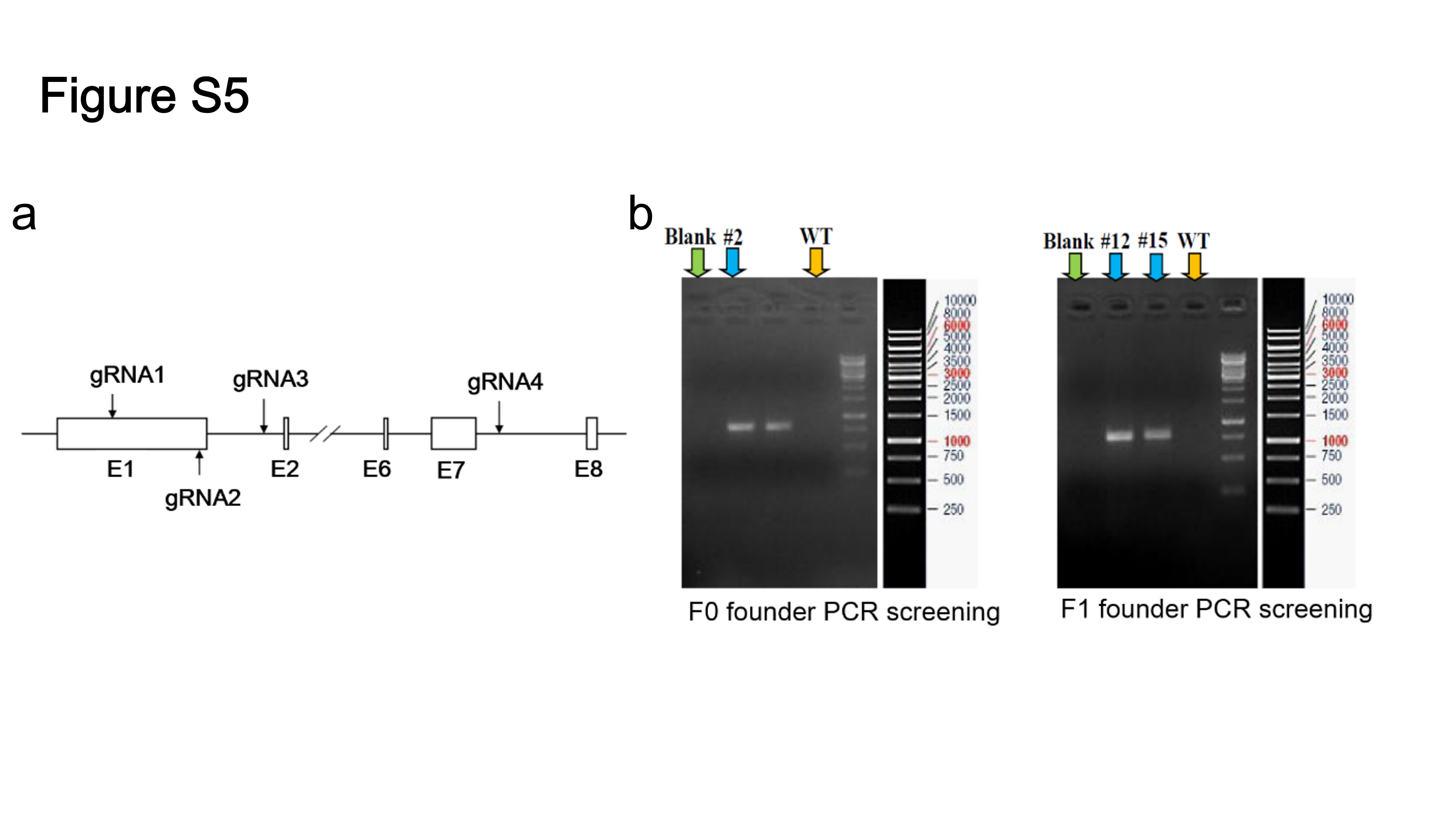

Supplement: Supplemental Material [file KGMI_A_2029997_SM9061.zip › Supplementary information/Supplementary Figure 5.tif]

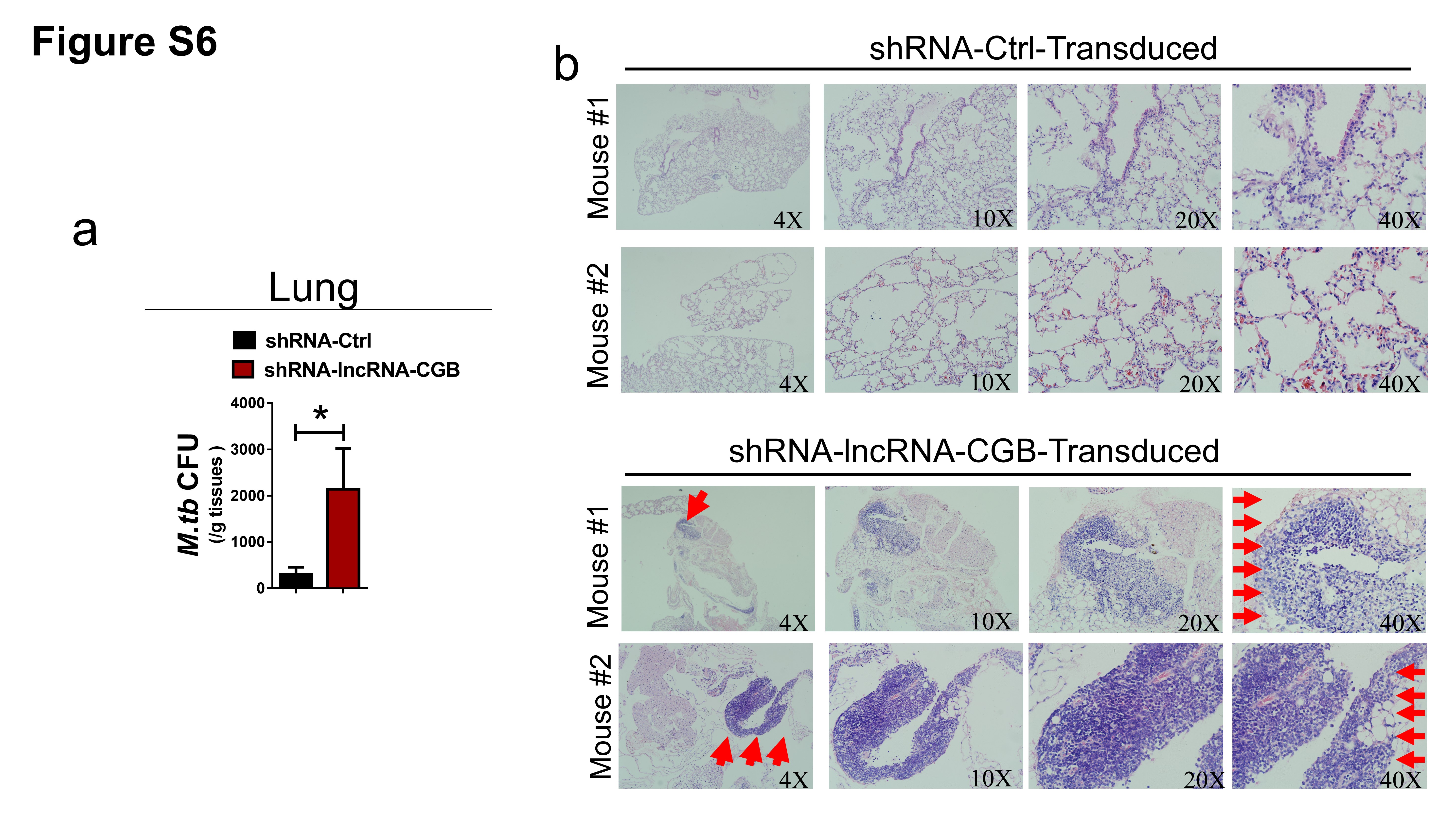

Supplement: Supplemental Material [file KGMI_A_2029997_SM9061.zip › Supplementary information/Supplementary Figure 6.jpg]

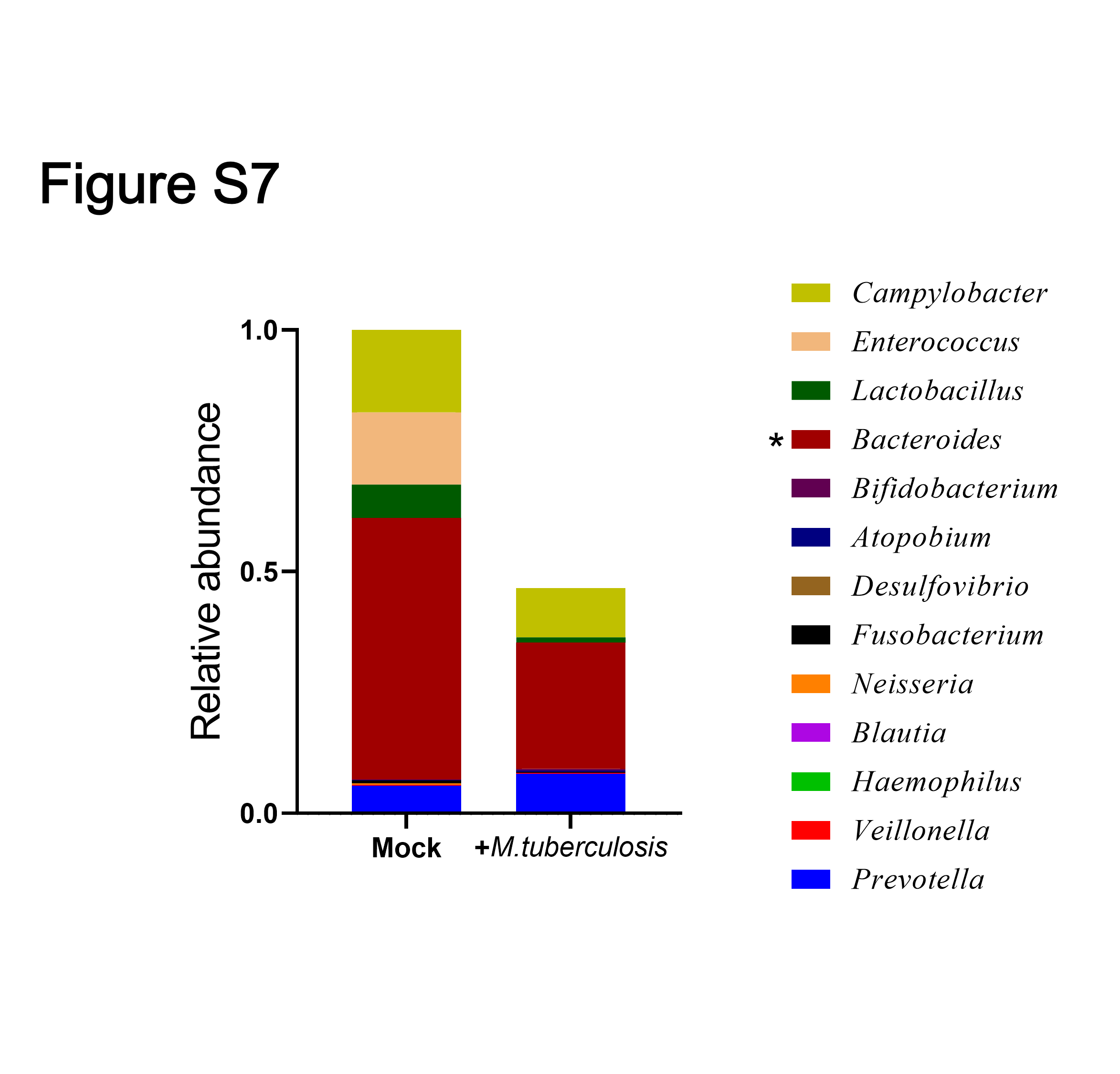

Supplement: Supplemental Material [file KGMI_A_2029997_SM9061.zip › Supplementary information/Supplementary Figure 7.tif]

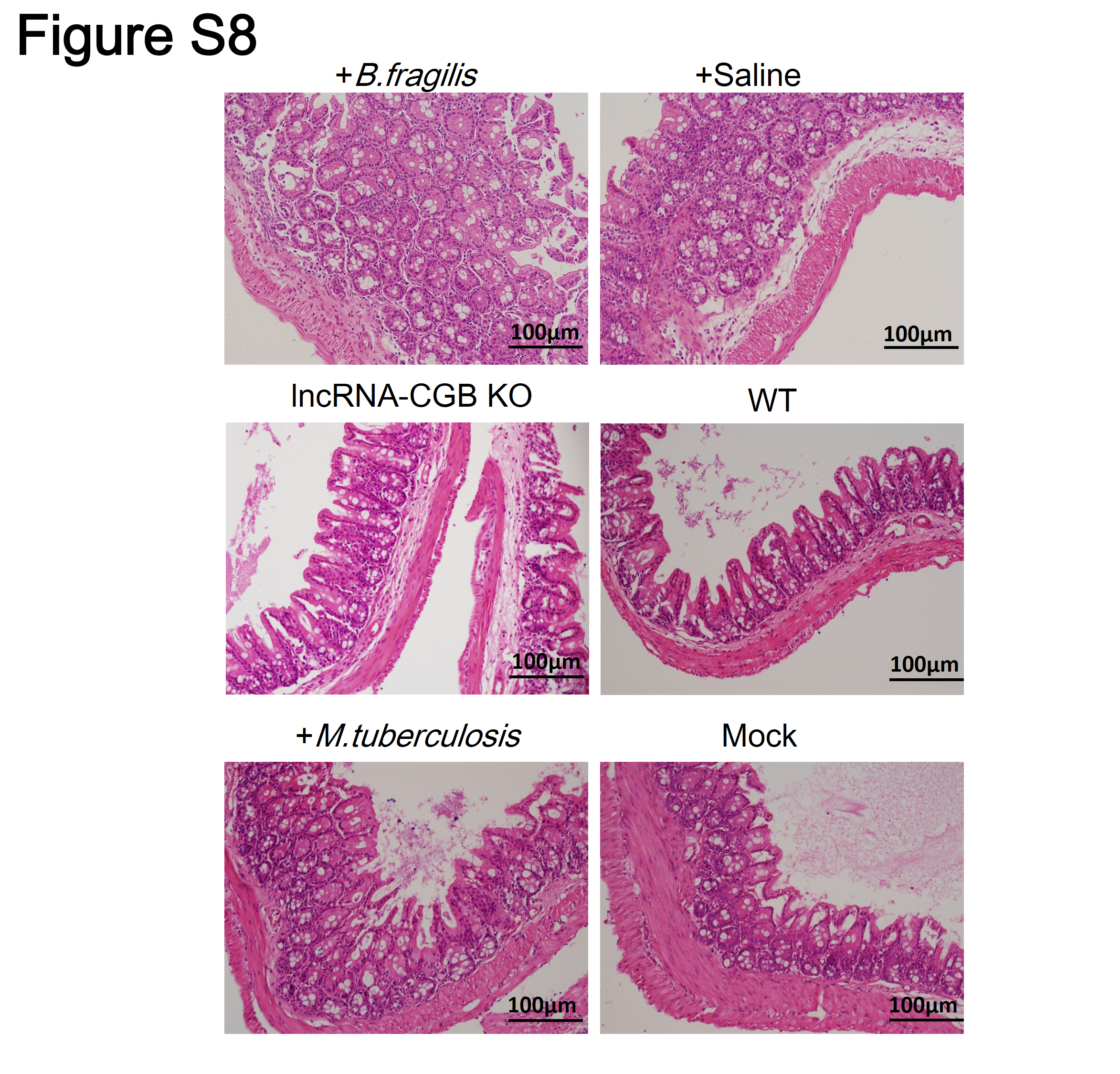

Supplement: Supplemental Material [file KGMI_A_2029997_SM9061.zip › Supplementary information/Supplementary Figure 8.TIF]

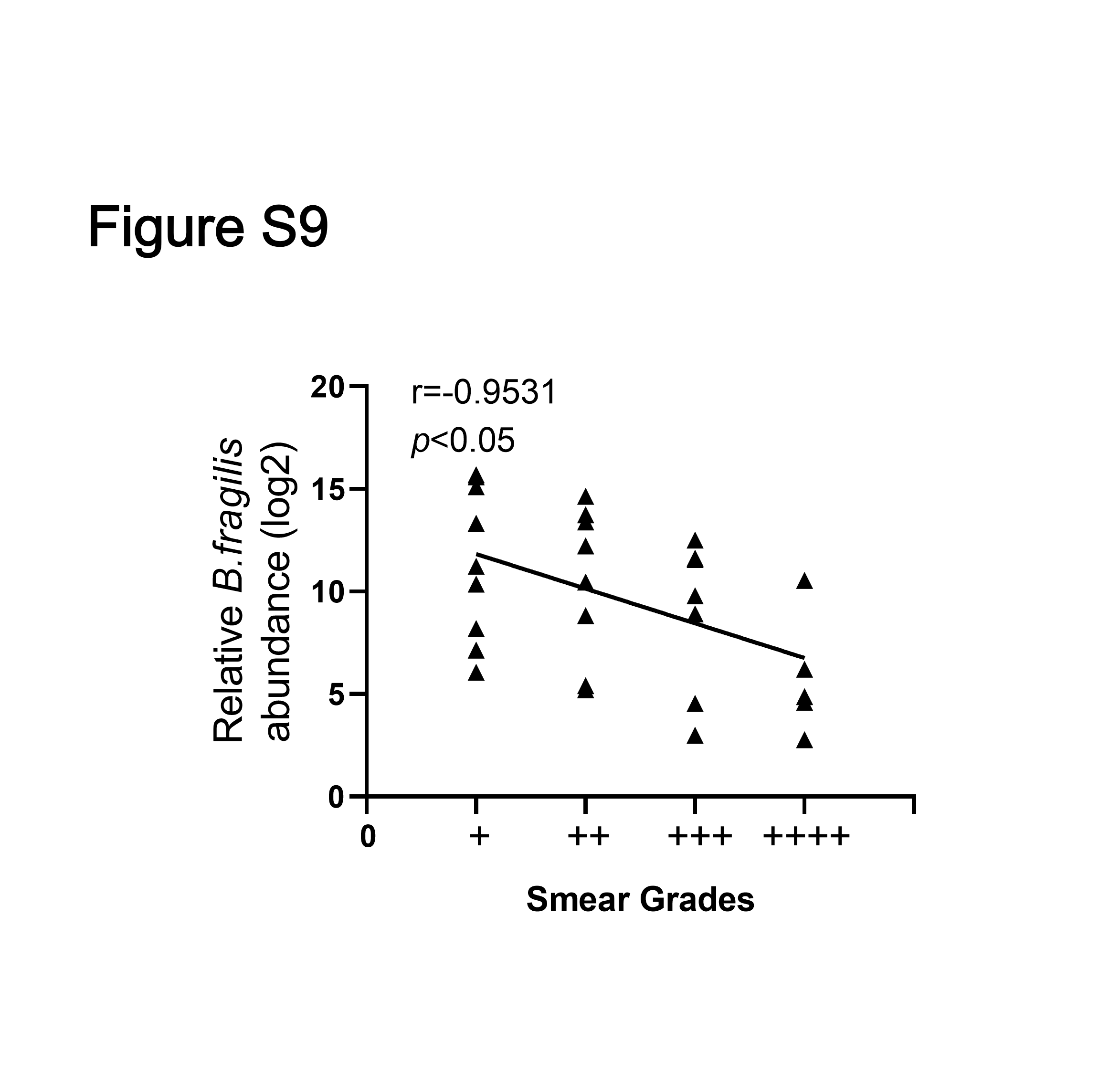

Supplement: Supplemental Material [file KGMI_A_2029997_SM9061.zip › Supplementary information/Supplementary Figure 9.tif]
